# scHi-CSim: a flexible simulator that generates high-fidelity single-cell Hi-C data for benchmarking

**DOI:** 10.1093/jmcb/mjad003

**Published:** 2023-01-27

**Authors:** Shichen Fan, Dachang Dang, Yusen Ye, Shao-Wu Zhang, Lin Gao, Shihua Zhang

**Affiliations:** School of Computer Science and Technology, Xidian University, Xi'an 710071, China; School of Automation, Northwestern Polytechnical University, Xi'an 710072, China; School of Computer Science and Technology, Xidian University, Xi'an 710071, China; School of Automation, Northwestern Polytechnical University, Xi'an 710072, China; School of Computer Science and Technology, Xidian University, Xi'an 710071, China; NCMIS, CEMS, RCSDS, Academy of Mathematics and Systems Science, Chinese Academy of Sciences, Beijing 100190, China; School of Mathematical Sciences, University of Chinese Academy of Sciences, Beijing 100049, China; Center for Excellence in Animal Evolution and Genetics, Chinese Academy of Sciences, Kunming 650223, China; Key Laboratory of Systems Health Science of Zhejiang Province, School of Life Science, Hangzhou Institute for Advanced Study, University of Chinese Academy of Sciences, Hangzhou 310024, China

**Keywords:** single-cell Hi-C data, simulator, high-fidelity, single-cell Hi-C clustering, distance-stratified sampling, benchmarking

## Abstract

Single-cell Hi-C technology provides an unprecedented opportunity to reveal chromatin structure in individual cells. However, high sequencing cost impedes the generation of biological Hi-C data with high sequencing depths and multiple replicates for downstream analysis. Here, we developed a single-cell Hi-C simulator (scHi-CSim) that generates high-fidelity data for benchmarking. scHi-CSim merges neighboring cells to overcome the sparseness of data, samples interactions in distance-stratified chromosomes to maintain the heterogeneity of single cells, and estimates the empirical distribution of restriction fragments to generate simulated data. We demonstrated that scHi-CSim can generate high-fidelity data by comparing the performance of single-cell clustering and detection of chromosomal high-order structures with raw data. Furthermore, scHi-CSim is flexible to change sequencing depth and the number of simulated replicates. We showed that increasing sequencing depth could improve the accuracy of detecting topologically associating domains. We also used scHi-CSim to generate a series of simulated datasets with different sequencing depths to benchmark scHi-C clustering methods.

## Introduction

The three-dimensional structures of chromosomes depict the spatial relationships and interactions between genes and regulatory elements in the genome, which are of vital importance to study gene regulation, cell fate decisions, evolution, and so on (Bonev and Cavalli, 2016). The chromatin conformation capture methods such as Hi-C give access to pairwise spatial interactions between genomic sites in a population of cells (Lieberman-Aiden et al., 2009; Dixon et al., 2012; Rao et al., 2014; Luo et al., 2021). After processing Hi-C data with corresponding computational methods (Crane et al., 2015; Durand et al., 2016; Zheng and Zheng, 2018; Ye et al., 2019b), previous studies have shown hierarchical structures of chromosomes, including A/B compartments, topologically associating domains (TADs), and chromatin loops.

In recent years, the rapid increase in the development of single-cell technologies has brought people's understanding of biological processes to the single-cell level (Nawy, 2014). For example, Liu et al. (2021) have profiled 103982 nuclei in the mouse brain by using single-nucleus DNA methylation sequencing. Yao et al. (2021) have profiled the single-cell transcriptomics and epigenomics in the mouse primary motor cortex. Among them, single-cell Hi-C (scHi-C) has been applied to probe the cell-to-cell variability and dynamics of chromatin structures among the cell population or across some critical biological processes (Nagano et al., 2013, 2017; Flyamer et al., 2017; Ramani et al., 2017, 2020; Stevens et al., 2017; Tan et al., 2018, 2019). The rapid promotion and application of scHi-C sequencing technologies has promoted the growth of scHi-C datasets. The original scHi-C dataset only involved dozens of cells (Nagano et al., 2013), while the later dataset could contain thousands of cells from different human cell lines (Ramani et al., 2017). Due to the limitations of experimental technology, the number of interactions in each cell was very inadequate. Later, Tan et al. (2018) innovated the experimental technique so that the number of interactions in a single cell could reach ˃1 million. Methyl-HiC (Li et al., 2019) and single-nucleus methyl-3C (Lee et al., 2019) sequencing technology can simultaneously capture chromatin organization and DNA methylation information. Tan et al. (2021) used MALBAC-DT (Chapman et al., 2020) and Dip-C (Tan et al., 2018) to generate transcriptome and three-dimensional genome of the developing mouse cortex and hippocampus after birth. Due to the high experimental cost, the techniques described above have not been widely used in complex tissues and different species. There exists a scHi-C dataset for thousands of cells with sufficient interactions originating from mouse embryonic stem cells (ESCs) throughout the cell cycle (Nagano et al., 2017). However, such qualified datasets are still rare, and due to the high cost, the corresponding replicate data are also lacking. Taken together, the high requirements for experimental technology and cost hinder the generation of scHi-C datasets for plenty of cells with ample sequencing depth and corresponding replicates for downstream analysis.

With the generation and accumulation of data, as well as the in-depth study of the three-dimensional structures of chromatin at the single-cell level, an increasing number of methods have emerged for the analysis of scHi-C data, such as the imputation, denoising, and embedding of scHi-C data, cell clustering, single-cell level chromatin structure identification, and three-dimensional modeling of chromatin structures (Liu et al., 2018; Zhou et al., 2019; Zhu and Wang, 2019; Kim et al., 2020; Han et al., 2021; Li et al., 2021; Yu et al., 2021; Zhang et al., 2021; Zheng et al., 2021). For example, Zhang et al. (2021) proposed Higashi for imputing scHi-C data, Yu et al. (2021) proposed SnapHiC to identify chromatin loops from scHi-C data, and Li et al. (2021) proposed DeTOKI to identify TAD-like domains in a single cell. However, high-quality and diverse datasets are required as the basis for performance evaluation and comparison during algorithm development. For example, evaluating methods for clustering and imputation of scHi-C data by multiple replicates with varied sequencing depths can achieve more reliable results. Because the experiments generated a very limited number of scHi-C data, it is necessary to generate high-fidelity simulation data according to the original data. To evaluate the downstream analysis methods, the simulator should be able to set the sequencing depth and cell number flexibly.

Now, generating simulated scHi-C data from bulk Hi-C or pooled scHi-C data by random partition or downsampling are prevalent approaches (Zhou et al., 2019). However, these methods don't take the inherent heterogeneity in scHi-C data into consideration during the simulation process and they do not explore whether the simulation data can maintain the multi-scale chromatin structure features contained in the original data. Reconstruction of three-dimensional chromatin structures with scHi-C data and disturbing the reconstructed models to generate pseudo-replicates are recognized strategies for studying imputation effects (Han et al., 2021). But these simulation methods highly depend on the accuracy of the three-dimensional modeling results, the unstable structures will lead to unreliable simulation data. In addition, there are some simulation methods designed for bulk Hi-C data. For example, Sim3C (DeMaere and Darling, 2018) generates simulated data with preset virtual chromatin structures and contact distribution learned from the real Hi-C data. FreeHi-C (Zheng and Kele, 2020) generates simulated data by following the standard Hi-C experimental protocol to sample fragment interactions (FIs). However, the severe sparsity and inherent heterogeneity in scHi-C data prevent the direct application of these methods to scHi-C data.

To this end, we developed a scHi-C simulator (scHi-CSim), which enables the generation of high-fidelity scHi-C data with the adjustable replicate number, cell number, and sequencing depth for each cell. We demonstrated that the simulated scHi-C data generated by scHi-CSim can capture the characteristics of the real data and maintain the multi-scale structural features. Furthermore, it can be a useful and valuable tool for benchmarking methods designed for scHi-C data analysis.

## Results

### Overview of scHi-CSim

**Figure 1:**
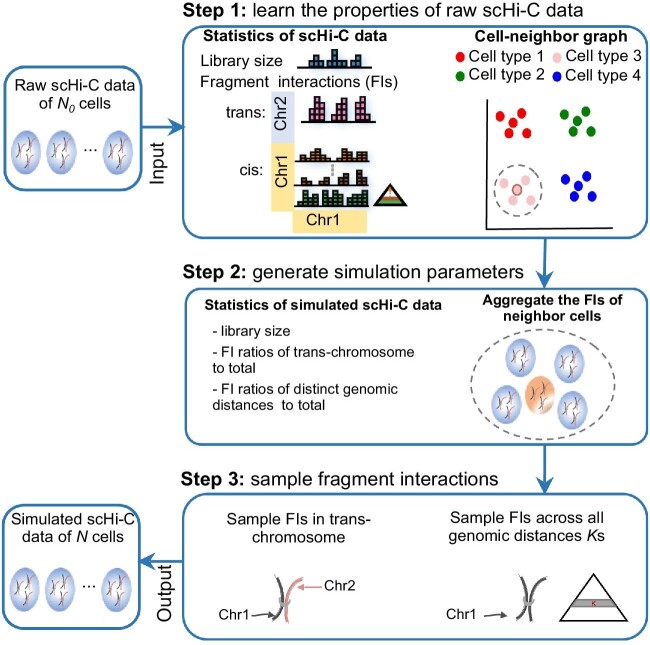
Illustration of the workflow of scHi-CSim. The whole process of scHi-CSim includes the following three steps. Step 1: after inputting raw data, scHi-CSim counts the library size, the FIs of trans-chromosome, the FIs of different genomic distances of cis*-*chromosome, and constructs the cell-neighbor graph. Step 2: scHi-CSim uses interval sample to generate library size, FI ratios of trans-chromosome to total, and FI ratios of distinct genomic distances of cis-chromosome to total for simulated data. Besides, scHi-CSim aggregates the FIs of neighbor cells according to the cell-neighbor graph. Step3: scHi-CSim samples FIs in trans-chromosome and samples FIs across all genomic distances in cis-chromosome according to the simulation parameters. Finally, scHi-CSim merges the FIs of trans-chromosome and cis-chromosome together as the simulated scHi-C data.

scHi-CSim aims to generate simulated scHi-C data based on an original scHi-C dataset. To create high-fidelity simulation data, scHi-CSim consists of three key steps (Figure 1; Materials and methods). Step 1: learn the properties of raw scHi-C data; Step 2: generate simulation parameters; Step 3: sample FIs. In the default mode, scHi-CSim estimates sequencing depth from raw data to generate simulated data. Beyond that, scHi-CSim also accepts the specific sequencing depths provided by the user. Besides, scHi-CSim distributes the simulation for different cells on different processors to implement multi-kernel computation, which shortens time usage. The more detailed tutorials of scHi-CSim are available at GitHub (https://github.com/zhanglabtools/scHi-CSim).

### scHi-CSim simulates high-fidelity scHi-C data

The most basic requirement for simulating data is to maintain some basic characteristics of the original data. We demonstrated that the simulated scHi-C data generated by scHi-CSim capture the key properties of the original scHi-C data. Here, 1171 single cells (Nagano et al., 2017) obtained from the mouse ESCs were simulated and analyzed. We used CIRCLET (Ye et al., 2019a) to construct the cell-neighbor graph and generated the simulated data with the same sequencing depths as those of the original data.

**Figure 2:**
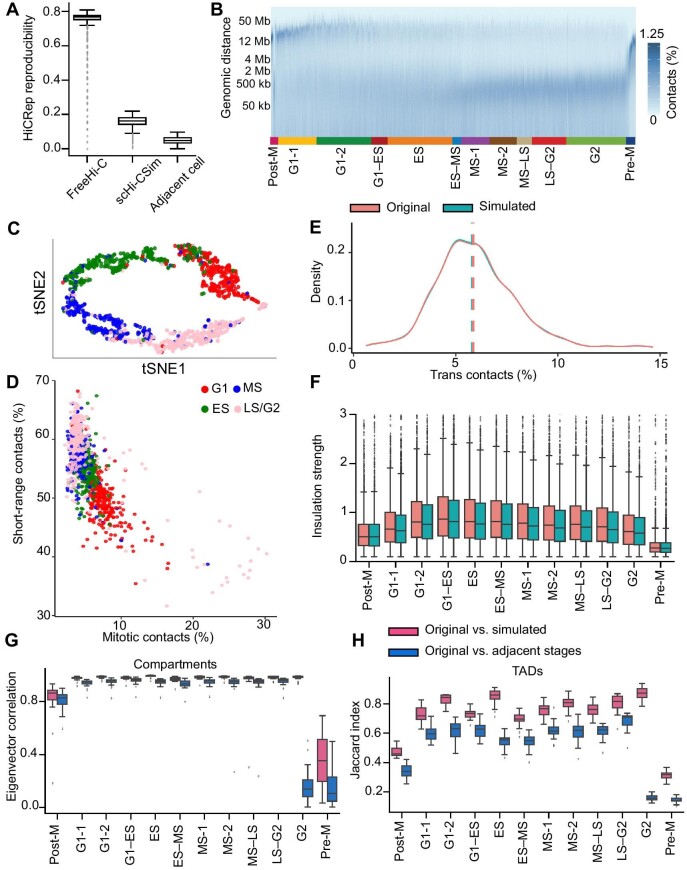
Basic properties of scHi-CSim-simulated data. (**A**) HiCRep reproducibility of the contact matrices (100 kb) between the original single-cell data and their FreeHi-C-simulated data, their scHi-CSim-simulated data and their adjacent cell data. (**B**) scHi-CSim-simulated single-cell contact decay profiles ordered by CIRCLET-inferred cell cycle phasing. Each column represents a single cell. (**C**) tSNE maps of scHi-CSim-simulated single cells calculated by CIRCLET based on the combination of feature sets CDD and PCC from four FACS-sorted cell phases (G1, ES, MS, and LS/G2). (**D**) Percentage of short-range (<2 Mb) versus mitotic band (2–12 Mb) contacts per cell, simulated by scHi-CSim. Cells are coloured by four FACS-sorted cell phases (G1, ES, MS, and LS/G2). (**E**) Percentage of trans-chromosomal contacts per scHi-CSim-simulated cell. Original single cells are exhibited in Supplementary material. The dashed lines represent medians. (**F**–**H**) The original scHi-C maps are pooled into 12 stages from CIRCLET phases. The correlated scHi-CSim-simulated scHi-C maps are pooled in the same way. (**F**) Insulation strength of TADs' boundaries. (**G**) Pearson correlation analysis of the A/B compartment eigenvector between the original pooled Hi-C maps and scHi-CSim-simulated pooled Hi-C maps, and the original pooled Hi-C maps of adjacent stages. The A/B compartment eigenvector is calculated by CscoreTool (100 kb). (**H**) Jaccard index of the TADs detected using the original pooled Hi-C maps and scHi-CSim-simulated pooled Hi-C maps, and the original pooled Hi-C maps of adjacent stages. TAD boundaries are detected using the Insulation Score (25 kb).

We first verified the necessity for aggregating cells by comparing the results of FreeHi-C (Zheng and Kele, 2020) and scHi-CSim. FreeHi-C is a customizable simulator by sampling FI pairs to generate Hi-C data according to empirical estimated frequencies. The sparse matrix of FIs per cell is taken as the input of FreeHi-C in the same manner with scHi-CSim to make the results comparable. Then, we measured the similarity between original data and simulated data with HiCRep, which is a measurement of reproducibility for Hi-C data by estimating the stratum-adjusted correlation coefficient (Yang et al., 2017). Using the values of HiCRep between adjacent cells as the baseline, the values between FreeHi-C-simulated and original cells are obviously too high, but the values between scHi-CSim-simulated and original cells are closer to the baseline, which indicates that the combination of neighbor cells is vital in overcoming the sparsity of the original scHi-C data (Figure 2A).

Additionally, since scHi-CSim samples cis-chromosomal FI pairs in distinct genomic distances, we exhibited the indispensable role of this procedure in maintaining heterogeneity of scHi-C data by computing decay profiles (Nagano et al., 2017), which are the ratios of contacts in distinct genomics to the total of each cell. For simplicity, this procedure is named distance-stratified sampling. Compared with the original cells (Supplementary Figure S2A), the decay profiles of scHi-CSim-simulated cells performing distance-stratified sampling (Figure 2B) is more similar than the decay profiles of scHi-CSim-simulated cells not performing distance-stratified sampling (Supplementary Figure S3). This shows that distance-stratified sampling enhances the ability of scHi-CSim to maintain heterogeneity of the original scHi-C data.

The scHi-C data were labelled with four cell cycle stages. To assess whether scHi-CSim is able to recapitulate the cell cycle stages, we used a cell cycle stage inferring method CIRCLET (Ye et al., 2019a) to classify the scHi-C data and embed the results with tSNE. The similar embedding results between scHi-CSim-simulated data and original data show that scHi-CSim is able to capture the category characteristics of the original data (Figure 2C; Supplementary Figure S2B). A previous study (Nagano et al., 2017) showed that the percentage of short-range ($ < $2 Mb) contacts and mitotic band (2–12 Mb) contacts per cell were important to characterize the cell stage. Here, we demonstrated that scHi-CSim can successfully keep the percentage of short-range and mitotic band contacts (Figure 2D; Supplementary Figure S2C). Besides, we also confirmed that the distribution of trans-contact percentages is extremely consistent between original and simulated data (Figure 2E).

Previous studies have showed that there exist many higher-order structures in the chromosomes, such as the compartments and TADs (Lieberman-Aiden et al., 2009; Dixon et al., 2012), but the sparsity of scHi-C data impedes the detection of the compartments and TADs. Therefore, we pooled the cells among the same stages (Figure 2B bottom) together to produce the merged Hi-C data for the detection of structures. The compartments were detected with CscoreTool (Zheng and Zheng, 2018), a statistical model outputting eigenvectors ranging between –1 and 1 to represent the A/B compartments. The results of the eigenvectors correlation (here we used Pearson correlation coefficient) between original and simulated data showed that scHi-CSim successfully preserved the compartments (Figure 2G). The calling of TADs is based on Insulation Score (Crane et al., 2015), a measurement of the extent to which a locus is not in a locally densely interacting area. Here, we used Juicer (Durand et al., 2016) to convert the FI matrices to the fixed-bin matrices for the detection of TADs. The evaluation of the results of TADs was implemented by calculating the Jaccard index between original and simulated TAD sets. More specifically, the ratio of the overlapping boundaries between original and simulated TAD sets to the total boundaries of original and simulated TAD sets is defined as the Jaccard index of TAD sets. The Jaccard index between original and simulated cells also reveals that the high-structures, such as TADs, are inhibited in the simulated data (Figure 2H). Heatmaps of Chromosome 11 of the merged Hi-C data generated by scHi-CSim is high-fidelity compared with the original Hi-C data (Supplementary Figure S1). As the structures of the Pre-M stage and the Post-M stage are not obvious, the detection of the compartments or TADs in the two stages is not stable and the corresponding eigenvector correlation and Jaccard index are lower than others. Besides, we also showed that the Insulation Strength, a measurement for the extent of difference between the two sides of the TAD boundaries, is conserved in simulated data (Figure 2F). In summary, scHi-CSim has the ability to maintain the critical characteristics of the original data and generates high-fidelity scHi-C data.

### scHi-CSim preserves cell type information of the original data

We tested four cell-neighbor graph construction methods including the principal component analysis (PCA), scHiCluster (Zhou et al., 2019), HiCRep/multidimensional scaling (MDS) (Liu et al., 2018), and latent Dirichlet allocation (LDA) (Kim et al., 2020) on two datasets (Ramani et al. dataset and 4DN sci-Hi-C dataset). The cell types of the current two datasets are known. In future, with the development of scHi-C sequencing technology, more and more datasets with unknown cell types will appear, such as scHi-C data of brain tissue (Tan et al., 2021). Therefore, we constructed the cell-neighbor graph with and without cell type information, respectively. The cell-neighbor graph is constructed on each cell type separately if we use cell type information. Otherwise, it is constructed on all cell types. Then, we generated the simulated data with the same sequencing depths as those of the original data.

**Figure 3:**
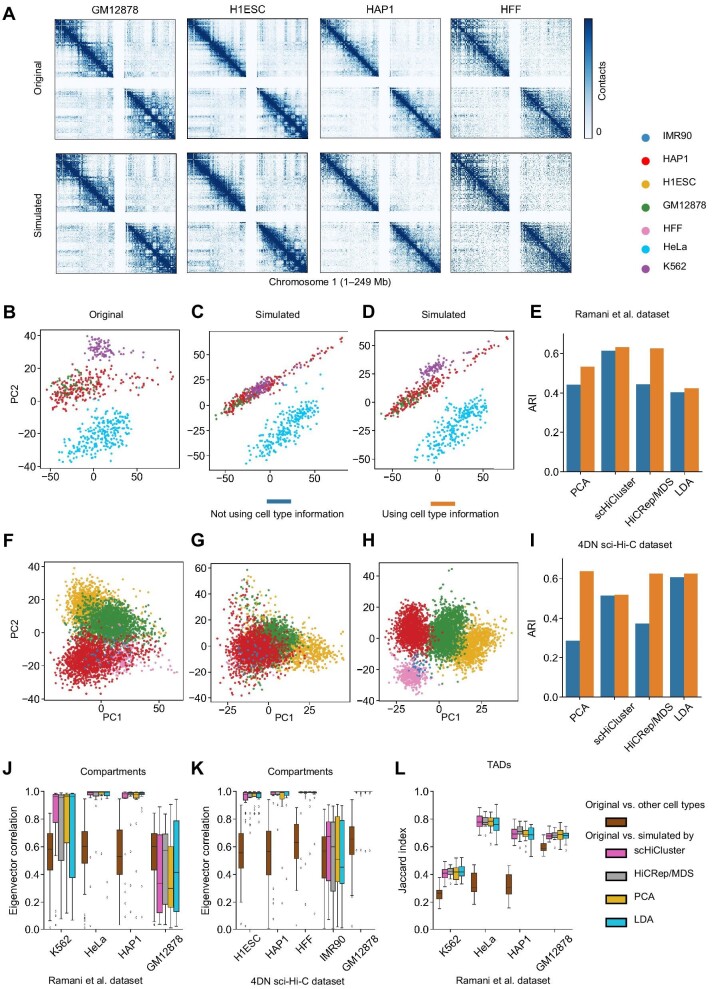
Key properties of simulated data based on the Ramani et al. dataset and 4DN sci-Hi-C dataset. (**A**) The original and simulated pooled scHi-C heatmap of four cell types came from 4DN sci-Hi-C dataset in chromosome 1 (500 kb). When generating the simulated data, scHi-CSim uses PCA to construct the cell-neighbor graph and uses cell type information. (**B**–**D**) The clustering result of scHiCluster on the original (**B**) and simulated (**C** and **D**) Ramani et al. dataset. (**F**–**H**) The clustering result of scHiCluster on the original (**F**) and simulated (**G** and **H**) 4DN sci-Hi-C dataset. The embedding of scHiCluster clustering results on simulated data in **C** and **G** use PCA for constructing cell-neighbor graphs and do not use cell type information. The embedding of scHiCluster clustering results on simulated data in **D** and **H** use PCA for constructing cell-neighbor graph and use cell type information. (**E** and **I**) ARI of scHiCluster clustering results on simulated datasets is generated with four methods for constructing cell-neighbor graphs. Each cell-neighbor graph method has two strategies: using cell type information and not using cell type information. (**J** and **K**) Pearson correlation analysis of the A/B compartment eigenvector between the original pooled scHi-C maps and scHi-CSim-simulated pooled scHi-C maps, and the original pooled scHi-C maps of other cell types. The simulated data use four methods for constructing cell-neighbor graphs and use cell type information. The A/B compartment eigenvector is calculated by CscoreTool (500 kb). (**L**) Jaccard index of the TADs detected using the original pooled scHi-C maps, scHi-CSim-simulated pooled scHi-C maps, and the original pooled scHi-C maps of other cell types. TAD boundaries are detected using the Insulation Score (25 kb for K562, HeLa, and HAP1; 100 kb for GM12878).

In order to uniformly evaluate the impact of different strategies for constructing close proximity graphs on the simulation data, we used scHiCluster to cluster the simulation data (Figure 3B–I). We noticed that using cell type information would improve the clustering effect (Figure 3E and I). For example, for PCA, a more general clustering method, there is no specific design for scHi-C data, and the classification of different cell types is not accurate enough (Supplementary Figure S4). When using cell type information, K562 cells and HAP1 cells are separated correctly in the Ramani et al. dataset (Figure 3D), and GM12878 cells, H1ESC cells, HAP1 cells, and HFF cells are classified more clearly in the 4DN sci-Hi-C dataset (Figure 3H). As scHiCluster and LDA can extract the characteristics of single-cell data more effectively than PCA (Supplementary Figure S4), the use of cell type information only slightly improved the clustering results (Figure 3E and I; Supplementary Figures S6 and S8 and Table S1). Cell type information is also useful for HiCRep/MDS despite the fact that HiCRep/MDS is designed for periodic cell data, and not suitable for the Ramani et al. dataset and 4DN sci-Hi-C dataset (Figure 3E and I; Supplementary Figure S7 and Table S1). Therefore, the use of cell type information can make the cell type characteristics of the simulation data more accurate. When there is no cell type information, we need to choose a more suitable neighbor graph construction method for different data sets. For example, scHiCluster is more suitable for the Ramani et al. dataset, and LDA is more suitable for the 4DN sci-Hi-C dataset, respectively (Supplementary Figure S4).

We also compared the high-order structures between original data and simulated data. We combined cells of each cell type to form the pseudo-bulk data (Figure 3A) and detected compartments and TADs for the pseudo-bulk data. There are big differences between the compartments of different cell types, and the simulation data can well maintain the compartment structure of the original data (Figure 3J and K). GM12878 cell line of the Ramani et al. dataset and IMR90 cell line of the 4DN sci-Hi-C dataset only have 39 and 34 cells, respectively, which makes the detection of the compartments not accurate. Similarly, the simulated data can also maintain the TADs of the original data and the insulation strength of the boundary (Figure 3L; Supplementary Figure S9). In summary, scHi-CSim was able to maintain the characteristics of the cell types of the original data.

### scHi-CSim preserves biological functions and enhances the contacts of loops and differential loops

We used CIRCLET to construct the cell-neighbor graph and generated the simulated data with the same sequencing depths as those of the original data. The detection of loops was based on HiCCUPS of Juicer (Durand et al., 2016). The scHi-C data were divided into 12 stages (Figure 2B bottom) according to CIRCLET (Ye et al., 2019a). As the pooled Hi-C maps used in detecting compartments and TADs are too sparse to detect loops at 25 kb resolution, we generated five substages by merging similar substages. These substages include G1 (G1-1 and G1-2), ES, MS (MS-1 and MS-2), MS–G2 (MS–LS and LS–G2), and G2.

**Figure 4:**
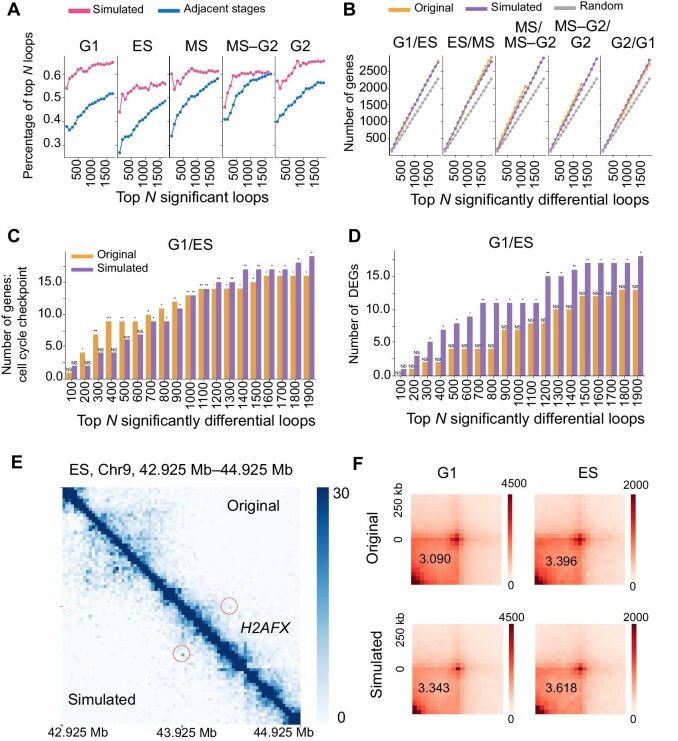
Performance of simulated data generated by scHi-CSim when detecting loops and differential loops. The single-cell Hi-C data of mouse ESCs containing cell cycle labels were divided into five subcycles. These subcycles include G1 (G1-1 and G1-2), ES, MS (MS-1 and MS-2), MS–G2 (MS–LS and LS–G2), and G2. Loop detection is performed by HiCCUPS. Differential loop detection is performed by HiCCUPS Diff. (**A**) Precision computed as the percentage of top $N$ ($N = 100,200, \cdots $) significant loops of simulated data or adjacent stage data of subcycles that are in the Top $N$ raw set. The common loops are defined with Euclidean distance of two loops less than the specific threshold (offset 5, threshold 7.07) in 25 kb Hi-C map. (**B**) The number of genes in Top *N* significant differential loops of raw data, scHi-CSim-simulated data, and random data. (**C** and **D**) Hypergeometric testing for significant analysis of genes associated with cell cycle checkpoint (**C**) and DEGs (**D**). NS, not significant; $P > 5 \times {10}^{ - 2}$; *$P < 5 \times {10}^{ - 2}$; **$P < 5 \times {10}^{ - 3}$; ***$P < 5 \times {10}^{ - 4}$. (**E**) The location of loop corresponding to gene *H2AFX* in G1 stage. (**F**) APA of G1 and ES. The juicer apa is used for performing APA.

Firstly, to evaluate the loop results of the simulation data, we calculated and compared the percentage (see Supplementary material) of simulated data to that of the original data. The loops were sorted according to false discovery rate (FDR) generated by HiCCUPS. The significant loops from top 100 to 2000 in the simulated data are very consistent to those of the original data (Figure 4A) compared with the adjacent stages.

Next, we detected the differential loops between adjacent stages by HiCCUPS Diff of Juicer for the analysis of cell cycle-associated genes. The number of genes overlapping with differential loops from top 100 to 2000 is consistent between raw and simulated cells (Figure 4B), in which the differential loops were sorted according to FDR generated by HiCCUPS Diff. Notably, only 1207 differential loops were found between MS and MS–G2 of raw data, and 1305 differential loops were found between MS–G2 and G2 of raw data. We performed the hypergeometric test (see Supplementary material) for the appearance of cell cycle-annotated genes and the differentially expressed genes (DEGs), in which the DEGs were detected with DESeq2 (Love et al., 2014). We found that the numbers of genes related to cell cycle checkpoint, genes related to cell cycle process and regulation, and DEGs are similar between raw and simulated cells (Figure 4C and D; Supplementary Figure S10). These results reveal that the procedures of scHi-CSim have successfully retained the chromosomal interactions correlated with gene regulation.

Besides, scHi-CSim can increase the contacts of loops to make them easier to detect. For example, one differential loop associated with one cell cycle checkpoint gene, *H2AFX*, is strengthened significantly in simulated data (Figure 4E). To verify that this phenomenon is widespread, we used aggregate peak analysis (APA) (Durand et al., 2016), an aggregating analysis of a loop set, to evaluate the total significance of the common loops between raw and simulated data. Then, we used peak to lower left (P2LL), the ratio of the central pixel to the mean of the pixels in the lower-left corner, to quantify the results of APA, and found that the P2LL values of the simulated data are higher than the raw (Figure 4F; Supplementary Figure S11). Overall, scHi-CSim could well capture the functional loops and enhance their statistical significance.

### Increasing sequencing depth enhances the accuracy of detecting high-order structures of chromosomes

**Figure 5:**
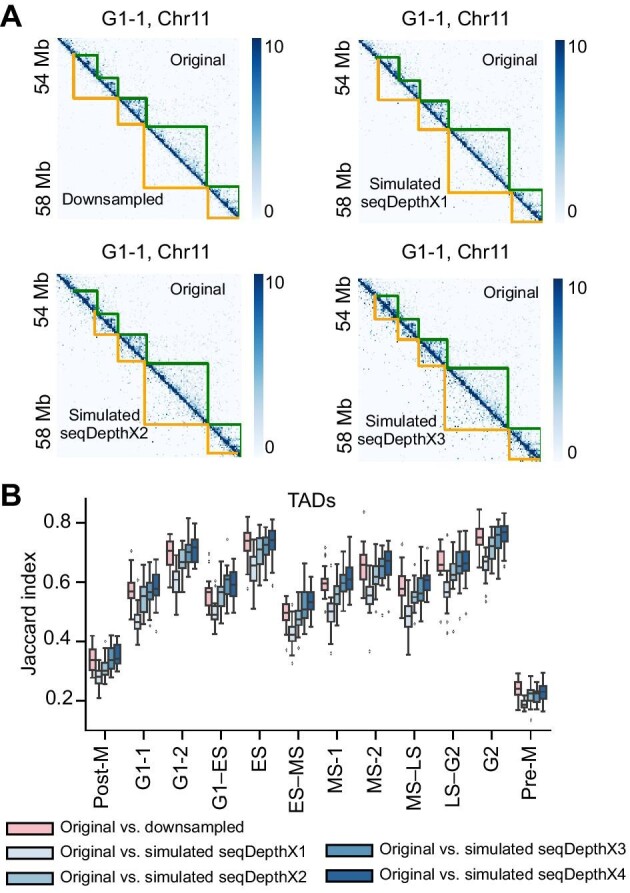
Accuracy of TAD detection with the increasing sequencing depth. (**A**) Comparison of Hi-C heatmaps between raw data and downsampled or simulated data with distinct sequencing depths. The green lines indicate the position of TADs called from raw Hi-C map, and the yellow lines indicate the position of TADs called from downsampled or simulated data. (**B**) Jaccard index of the TADs detected using the raw pooled Hi-C maps and the other five situations: downsampled raw Hi-C maps, simulated Hi-C maps with 1×, 2×, 3×, and 4× sequencing depths.

Due to the high experimental cost, it is difficult to generate data with different sequencing depths through biological experiments to unveil the relationship between sequencing depth and the accuracy of compartment and TAD detection. scHi-CSim can flexibly generate simulated data with diverse sequencing depths by adjusting parameters. Therefore, to assess the effects of sequencing depth on the detection of high-order structures, we first downsampled the Nagano et al. dataset to 25% sequencing depth per cell and applied scHi-CSim onto such downsampled data to generate simulated data. We increased the sequencing depth of simulated data from 100% to 400% per cell and then detected compartments (with CscoreTool) (Zheng and Zheng, 2018) and TADs (with Insulation Score) (Crane et al., 2015) in pooled data according to cell cycle stages (Ye et al., 2019a). Intuitively, the visualization of TADs was significantly improved after increasing sequencing depth (Figure 5A). We compared the TADs detected in raw data between downsampled and simulated data, and found that the consistency between TAD sets is enhanced with the increasing sequencing depth in terms of Jaccard index (Figure 5B). As to the compartment detection, detection on the simulated data keeps the similar effectiveness compared with that on the downsampling data (Supplementary Figure S12). In summary, the simulated data of scHi-CSim reveal that the compartments are stable in low sequencing depth data, and increasing sequencing depth will improve the accuracy of TADs detection. This experiment displayed the ability of scHi-CSim for generating benchmarking dataset.

### scHi-CSim benchmarks clustering methods by generating a series of simulated data with different sequencing depths

**Figure 6:**
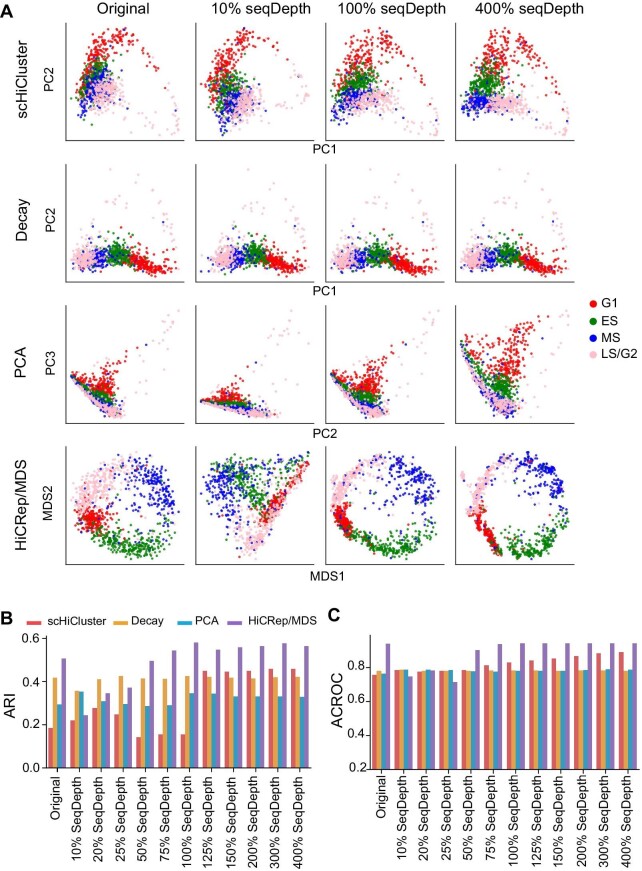
Performance evaluation of scHiCluster, Decay, PCA, and HiCRep/MDS on the scHi-CSim-simulated scHi-C data. (**A**) The embedding of simulated data with different sequencing depths. (**B**) ARI of clustering results with different sequencing depths. (**C**) ACROC of clustering results with different sequencing depths.

Here, we demonstrated the ability of scHi-CSim for benchmarking scHi-C clustering methods, which are used to study the differences in chromosome structure between different types of cells. We used scHi-CSim to generate simulated data with a series of sequencing depths based on the Nagano et al. dataset for benchmarking scHiCluster (Zhou et al., 2019), Decay (Nagano et al., 2017), PCA, and HiCRep/MDS (Liu et al., 2018). scHiCluster uses linear convolution and random walk for imputation and then uses PCA to get a low-dimensional representation of scHi-C data for clustering. Decay uses the proportion of contacts at each genomic distance to form the features of each cell and then clusters all cells in the same manner as scHiCluster. PCA directly uses the scHi-C data for clustering. HiCRep/MDS clusters all cells by calculating the similarity between each cell with HiCRep, converting the simialrity to distances and projecting the distances into two-dimensional space with MDS. Firstly, the clustering results were projected to two dimensions to display the effect of clustering visually (Figure 6A; Supplementary Figures S13–S16). Here, following the previous studies, we used PC1 and PC2 for scHiCluster and Decay, PC2 and PC3 for the results of PCA (Zhou et al., 2019), and MDS1 and MDS2 for HiCRep/MDS to get the best performance. We also used adjusted rand index (ARI), calculated circular ROC (CROC) score, and average CROC (ACROC) to assess the clustering performance. To evaluate the cell cycle data, the embedding of each stage is assumed to obey a circular normal distribution (Liu et al., 2018). Then, we calculated the CROC score and ACROC after fitting each stage to a circular normal distribution (see Supplementary material). Four CROC scores under the four stages were averaged to get ACROC (Figure 6C; Supplementary Figure S17).

The embedding results show that the change in sequencing depth has less impact on Decay but has a more significant effect on PCA (Figure 6A; Supplementary Figures S13 and S14). The embedding of PCA clustering results changes from compact to scattered as the depth of sequencing increases, but Decay almost stays the constant. Decay uses the percentages of interactions at each genomic distance as features, which makes it more robust to the changes of sequencing depth. Therefore, Decay is more suitable for low sequencing data than PCA. The ACROC results (Figure 6C) show that the increase in sequencing depth can enhance the ability of scHiCluster to classify cells in different phases. For example, as the sequencing depth changed from 125% to 400%, cells in MS phase and LS/G2 phase were more clearly classified (Supplementary Figure S15). HiCRep/MDS has a significant improvement in clustering effect as the sequencing depth increases from 10% to 100%, and a slight change in clustering effect from 100% to 400% (Figure 6A–C; Supplementary Figure S16). Therefore, HiCRep/MDS is well suited for data with cell cycles, while scHiCluster requires more interactions to enhance the clustering effect. In summary, scHi-CSim has the ability to benchmark scHi-C clustering analysis methods.

## Discussion

With the development of scHi-C sequencing technologies, a substantial fraction of scHi-C-seq analytical methods has gradually appeared in recent years. However, the shortage of simulation methods impedes the assessment of these methods with an impartial dataset. This prompted us to design scHi-CSim, a scHi-C data simulator that generates high-fidelity scHi-C data. To resemble the properties of biological scHi-C data, scHi-CSim uses interval sampling to mimic the library size, trans-chromosomal FIs, and cis-chromosomal distance-stratified FIs. scHi-CSim also utilizes the adjacent cells to exceed the limitation of single-cell sparsity. Furthermore, scHi-CSim accepts user-provided parameters to control the simulated data. scHi-CSim applies a flexible mode to assign each cell's library size and replicate numbers, which either are provided by users or learned from original data. The outputs of scHi-CSim are multiple FI matrices. scHi-CSim additionally provides scripts to merge and convert FI matrices to bin-interactions matrices with a specific resolution for downstream analysis.

We used the scHi-C dataset of mouse ESCs containing cell cycle substage labels to conduct a comprehensive analysis of scHi-CSim-simulated data. We demonstrated that the simulated data inherit primary characteristics from the biological data, such as visualization of Hi-C maps, decay profiles, clustering, and high-order chromatin structures. We also systematically compared the effects of different methods of constructing cell-neighbor graphs on simulation results using two human scHi-C datasets containing multiple cell types. Besides, the sequencing depth of simulated data is controlled by user-specific parameters, which enables scHi-CSim to generate simulated data with different sequencing depths. The evaluation of sequencing depth effects on the accuracy of the detection of chromatin high-order structures and scHi-C data clustering methods reveal the competence of scHi-CSim to generate benchmark datasets.

scHi-CSim is the first scHi-C data simulator that focuses on keeping the properties of biological data. Although scHi-C-seq technologies have proliferated in recent years, the high costs hinder the appearance of a biological benchmarking dataset. scHi-CSim allows users to adjust the library size and the number of cells to generate benchmarking datasets. This will make the evaluation of scHi-C analytical methods more convenient. Since scHi-CSim borrows information from similar cells, the quality of the simulation data relies on the accuracy of the cell proximity map. The users have to select methods carefully for the construction of the cell-neighbor graph. Although the sparsity of scHi-C data has a significant impact on the downstream analysis, as scHi-CSim samples fragment interactions, scHi-CSim is unable to control the sparsity of the data directly but could control the sequencing depth to affect the sparsity implicitly (Supplementary Figure S19). Given custom loops and TAD-like domains, scHi-CSim can be modified to contain high-order structures in simulated data for benchmarking. We expect scHi-CSim will assist the evaluation and promote the development of analytical methods.

## Materials and methods

### Datasets

In this section, we describe the datasets used in our experiments. These datasets include the Ramani et al. dataset (Ramani et al., 2017), 4DN sci-Hi-C dataset (Kim et al., 2020), and Nagano et al. dataset (Nagano et al., 2017). The Ramani et al. dataset includes four human cell lines (HeLa S3, HAP1, K562, and GM12878) and the 4DN sci-Hi-C dataset includes five human cell lines (GM12878, H1ESC, HAP1, HFF, and IMR90). The Ramani et al. dataset and 4DN sci-Hi-C dataset were mapped to the hg19 assembly. The processed Ramani et al. dataset is available at GSE84920 and the processed 4DN sci-Hi-C dataset is available at sci-Hi-C-site. We filtered the cells whose contact is ˂5 kb in the Ramani et al. dataset and 4DN sci-Hi-C dataset, and the number of cells used in our work is displayed in Supplementary Table S3. The Nagano et al. dataset contains 1992 diploid cells of mouse ESCs grown in 2i media which were filtered by stringent quality control. Specifically, 393506 restriction fragments and 127233 distinct contacting pairs as median coverage per cell were supplied by this dataset. The Nagano et al. dataset is available at the Gene Expression Omnibus under accession GSE94489 and Nagano et al. provided scHiC2, a corresponding processing tool (Nagano et al., 2017). After processed with scHiC2, the fastq files of the Nagano et al. dataset were converted to FI matrices, which are also available at the website of scHiC2. To evaluate the effect of scHi-CSim, we used 1171 cells of the Nagano et al. dataset, which are labelled with fluorescence-activated cell sorting (FACS) to four cell cycle stages. According to the results of FACS, the numbers of cells in ‘G1’ phase, ‘early-S’ phase, ‘mid-S’ phase, and ‘late-S/G2’ phase are 280, 303, 262, and 326, respectively. Furthermore, these cells are divided into 12 stages (Figure 2B bottom) by pseudo-trajectory reconstruction (Ye et al., 2019a). As the sparsity of scHi-C data impedes the detection of the compartments, TADs, and loops, we pooled the scHi-C data together to generate 12 Hi-C maps for the identification of compartments and TADs, or five Hi-C maps with more cells for the identification of loops in our following experiments.

A single-cell RNA-seq data of mouse ESCs (Buettner et al., 2015) was obtained from the European Bioinformatics Institute. The single-cell RNA-seq data contain 182 cells, including 59, 58, and 65 cells that belong to ‘G1’ phase, ‘S’ phase, and ‘G2M’ phase, respectively.

Besides, we obtained two lists of genes, which are highly related to cell cycle, from mouse genome informatics. The first list includes 1609 genes relevant to cell cycle process and regulation and the second list includes 168 genes relevant to cell cycle checkpoint.

### Methods

Given a scHi-C dataset with ${N}_0$ cells, our goal is to generate a new scHi-C dataset with $N$ cells (Figure 1). For each cell $q$, we denote its number of simulated cells as ${n}_q$, then $N = \mathop \sum _{q = 1}^{{N}_0} {n}_q$.

The input data of scHi-CSim are sparse matrices of genomic FIs per cell, in which the genomic fragments come from the experimental restriction enzyme cutting sites (see HiC-Pro manual for more details). Correspondingly, we define the set of FI number for cell $q$ as $X( q ) = {\{ {{x}_{mn}( q )} } \},m,n = 1, \cdots ,G$, where $G$ represents the number of all genomic fragments, and ${x}_{mn}( q )$ represents the number of interactions between fragments $m$ and $n$.

#### Step 1: learn the properties of raw scHi-C data

In this step, we construct a cell-neighbor graph for the aggregation of adjacent cells and learn the attributes of scHi-C data for obtaining the parameters of simulated cells.

First, we extract features of the original scHi-C data to get the low-dimensional embedding of each cell. Then, we calculate the Euclidean distance between cells based on the embedding and construct the cell-neighbor graph. Different clustering methods adopt diverse feature extraction methods. scHiCluster (Zhou et al., 2019) uses linear convolution and random walk (with start) to smooth scHi-C contact maps. After smoothing, scHiCluster keeps the top 20% interactions and projects the processed matrix into a low-dimensional space by PCA. HiCRep/MDS (Liu et al., 2018) uses HiCRep (Yang et al., 2017) to measure the cell similarity by combing each genomic distance and projects the cell similarity into two dimensions with MDS. Kim et al. (2020) converted scHi-C contact maps to cell-locus pair matrix, in which each entry represents one locus pair, and used LDA to decompose cell-locus pair matrix into cell-topic and topic-locus-pair matrix. Then, the cell-topic matrix is projected into a low-dimensional embedding space with UMAP.

CIRCLET (Ye et al., 2019a) shows that both contact probability distribution versus genomic distance (CDD) and pairs’ contact coverage (PCC) are useful feature sets for classifying cell cycle scHi-C data. CDD extracts the proportion of FIs at distinct log2 genomic distances. Firstly, fragment pairs of scHi-C data are assigned to specific bins with the following formula,


$$\begin{eqnarray*}{\rm{bin}}\left( d \right) = {\rm{floor}}\left( {\frac{{{\rm{lo}}{{\rm{g}}}_{\rm{2}}\left( d \right) + s}}{s}} \right),\end{eqnarray*}$$

where ${\rm{floor}}$ means rounding down the value, $d$ indicates the genomic distance of fragment pairs and $s$ represents an exponent step of each bin, whose typical values are 0.04, 0.1, 0.125, 0.2, and 0.33. The default value of $s$ is 0.04. For one cell, the contact probability for each bin is generated by the number of fragment pairs related to this bin divided by the total number of fragment pairs. Assume that after ${\rm{lo}}{{\rm{g}}}_{\rm{2}}$ transformation, there are $K$ bins in total, and then the contact probability distributions of ${J}_0$ cells on $K$ bins will form a ${J}_0 \times K$ matrix.

PCC extracts the fragment pairs with high variance among all cells. We convert the cis-chromosomal FI matrix of a scHi-C data to a matrix $A$ with bins of a fixed width (e.g. 100 kb, 500 kb, and 1 Mb) for each chromosome, where ${A}_{i,j}$ represents the number of FIs (or a normalized score) between bins $i$ and $j$. Then, we define ${\rm{Value}}( {{A}_{i,j}} ) = \frac{{\mathop \sum \nolimits_{q = 1}^{{J}_0} {A}_{i,j,q}}}{{{J}_0}}$ for the selection of bin pairs, in which ${\rm{q}}$ means the $q$th cell. Then, ${\rm{Var}}( {{A}_{i,j}} )$, the variance of ${\rm{Value}}( {{A}_{i,j}} )$ across all single cells, is calculated and bin pairs with the top 99% of ${\rm{Var}}( {{A}_{i,j}} )$ are selected as significant ones. Suppose we have $P$ significant bin pairs, finally ${J}_0$ cells on $P$ bin pairs will form a ${J}_0 \times P$ matrix.

The two feature sets are merged to form a ${J}_0 \times ( {K + P} )$ matrix as the combined features. Then, the low-dimensional embedding is calculated from the combined features by performing PCA dimensionality reduction.

We compared the clustering results of scHiCluster, LDA, and HiCRep/MDS on the Ramani et al. dataset and 4DN sci-Hi-C dataset, respectively. scHiCluster and LDA show higher ARI than that of HiCRep/MDS (Supplementary Figure S4C and D), which suggests that scHiCluster and LDA are more suitable for scHi-C data of multiple cell types than HiCRep/MDS. A previous study demonstrated that HiCRep/MDS was more suitable for scHi-C data with cell cycle (Zhang et al., 2021), such as Nagano et al. dataset. CIRCLET was designed for the construction of cell cycle trajectories (Ye et al., 2019a). After extracting low-dimensional embedding, scHi-CSim estimates Euclidean distances between each cell on the low-dimensional space. Finally, the raw cells and their most adjacent cells are passed into Step 2.

On the other hand, we compute three attributes of all cells in the following, including the library size, the ratio of trans-chromosome FIs to total FIs, and the ratio of cis-chromosome FIs of distinct genomic distances to total.

The set of library size for all raw cells is expressed as $ {\{ {f( 1 ), \cdots ,f( q ), \cdots ,f( {{J}_0} )} } \}$, where $f( q )$ is the number of all FIs in cell $q$.

The ratio of trans-chromosome FIs to total in cell $q$ is denoted as


$$\begin{eqnarray*}{P}_{{\rm{trans}}}\left( q \right) = \frac{{{f}_{{\rm{trans}}}\left( q \right)}}{{f\left( q \right)}},\end{eqnarray*}$$

where ${f}_{{\rm{trans}}}( q )$ is the FI number of trans-chromosomes in cell $q$. Similarly, the set of all cells is expressed as $ {\{ {{P}_{{\rm{trans}}}( 1 ), \cdots ,{P}_{{\rm{trans}}}( {{J}_0} )} } \}$.

Then, we divide the genomic distance in the same manner as PCC and define the ratio of fragments interactions in bin $k$ to total in cell $q$ as


$$\begin{eqnarray*}{\rm{fcd}}{\left( k \right)}_q = \frac{{f\left( {k,q} \right)}}{{f\left( q \right)}},\end{eqnarray*}$$

where $f( {k,q} )$ is FI number in bin $k$ in cell $q$. The set of all cells is denoted as $ {\{ {{\rm{fcd}}{{( k )}}_1, \cdots ,\ {\rm{fcd}}{{( k )}}_{{J}_0}} } \}$. This is processed for $K$ bins. Finally, the attributes of all raw cells are passed into Step 2.

#### Step 2: generate simulation parameters

In this step, the parameters of one simulated cell is generated according to the cell-neighbor graph and the attributes of raw cells. Here, we mark cell $\hat{j}$ as the chosen raw cell, and $j$ as the corresponding simulated one.

Firstly, we prepare the FI pool for sampling in Step 3. The candidates of FIs are collected from cell $\hat{j}$ and its most adjacent cells. The combination of cell $\hat{j}$ and its neighbors is written as $\widehat {{j}_c}$. The neighbor cells are determined by the cell-neighbor graph constructed in Step 1. The default number of neighbor cells is 20 and a user-custom number is also available at the setting of scHi-CSim.

Given the combination of cells $\widehat {{j}_c}$, its set of FI number is denoted as


$$\begin{eqnarray*}X\left( {\widehat {{j}_c}} \right) = \mathop \sum \limits_{l \in N\left( {\hat{j}} \right)\cup\left[ {\hat{j}} \right]} w\left( {l,\hat{j}} \right)X\left( l \right),\end{eqnarray*}$$

where $N( {\hat{j}} )$ represents the neighbor cells of cell $\hat{j}$, and the weight $w( {l,\hat{j}} )$ is inversely proportional to the distance between cell $l$ and $\hat{j}$. $X( {\widehat {{j}_c}} )$ will be used in Step 3 for computing the empirical frequencies of FIs.

Secondly, we use interval sampling to obtain three attributes of the simulated cell: library size, ratio of trans-chromosome FIs to total, and ratios of FIs to total in distinct genomic distances. Here, we take the generation of the third attributes as an example to display the process of interval sampling. In the beginning, we prepare $ {\{ {{\rm{fcd}}{{( k )}}_{\hat{1}}, \cdots ,\ {\rm{fcd}}{{( k )}}_{\widehat {{J}_0}}} } \}$ for the generation of ${\rm{fcd}}{( k )}_j$ by interval sampling (Li and Li, 2019). Then, we sort the set and use ${\rm{fcd}}{( k )}_{( t )}$ to indicate the $t$th ${\rm{fcd}}$ value. After dividing the ${\rm{fcd}}$ set into $B$ intervals, the ${\rm{fcd}}{( k )}_j$ of cell $j$ will be sampled from one interval. The first interval is denoted as


$$\begin{eqnarray*}\left( { - \infty ,{\rm{fcd}}{{\left( k \right)}}_{\left( 1 \right)} + \frac{{{\rm{fcd}}{{\left( k \right)}}_{\left( {{J}_0} \right)} - {\rm{fcd}}{{\left( k \right)}}_{\left( 1 \right)}}}{B}} \right),\end{eqnarray*}$$

the $b$th interval ($1 < b < B$) is denoted as


$$
\begin{eqnarray*}&&\left( {{\rm{fcd}}{{\left( k \right)}}_{\left( 1 \right)} + \frac{{{\rm{fcd}}{{\left( k \right)}}_{\left( {{J}_0} \right)} - {\rm{fcd}}{{\left( k \right)}}_{\left( 1 \right)}}}{B}\left( {b - 1} \right),} \right. {{\rm{fcd}}{{\left( k \right)}}_{\left( 1 \right)}}\\
&&\left.{
+ \frac{{{\rm{fcd}}{{\left( k \right)}}_{\left( {{J}_0} \right)} - {\rm{fcd}}{{\left( k \right)}}_{\left( 1 \right)}}}{B}b} \right),\end{eqnarray*}$$

and the $B$th interval is denoted as


$$
\begin{eqnarray*}\left( {{\rm{fcd}}{{( k )}}_{( 1 )} + \frac{{{\rm{fcd}}{{( k )}}_{( {{J}_0} )} - {\rm{fcd}}{{( k )}}_{( 1 )}}}{B}( {B - 1} ), + \infty }\right).\end{eqnarray*}$$

We use an indicating value ${I}_{\hat{j}} = b$ to represent ${\rm{fcd}}{( k )}_{\hat{j}}$ belonging to the $b$th interval. The indicating value is used for matching all ${\rm{fcd}}( s )$ belong to $b$th interval and random sample one ${\rm{fcd}}$ for the simulated cell $j$. Specifically, ${\rm{fcd}}{( k )}_j$ is generated by sampling from the stratified ${\rm{fcd}}$ estimated from the raw data:$\ {\rm{fcd}}{( k )}_j \sim {\rm{Uniform}}( { {\{ {{\rm{fcd}}{{( k )}}_{\widehat {j^{\prime}}}:{I}_{\widehat {j^{\prime}}} = {I}_{\hat{j}},\widehat {j^{\prime}} = 1, \cdots ,{J}_0} } \}} )$. The above procedure is repeated across all $K$ bins for the generation of $ {\{ {{\rm{fcd}}{{( 1 )}}_j, \cdots ,\ {\rm{fcd}}{{( K )}}_j} } \}$.

Similarly, we generate $f( j )$ and ${P}_{{\rm{trans}}}( j )$ with the same procedure as ${\rm{fcd}}$. We use interval sampling for $ {\{ {f( 1 ), \cdots ,f( q ), \cdots ,f( {{J}_0} )} } \}$ and $ {\{ {{P}_{{\rm{trans}}}( 1 ), \cdots ,{P}_{{\rm{trans}}}( {{J}_0} )} } \}$, respectively. The user-custom FI number $f( j )$ is also accepted by reading from the user-provided file.

#### Step 3: sample FIs

For cell $j$, we sample FIs by the following procedures: (i) calculating the FI numbers of trans-chromosome and cis-chromosomal at distinct genomic distances; (ii) sampling FIs of trans-chromosome; (iii) sampling FIs across all genomic distances.

Let ${{\rm{\pi }}}_{mn}( {\widehat {{j}_c}} )$ represent the empirical frequencies of FIs in cell $\widehat {{j}_c}$. We have


$$
\begin{eqnarray*} {{\rm{\pi }}}_{mn} ( {\widehat {{j}_c}} ) = \frac{{{x}_{mn}( {\widehat {{j}_c}} )}}{{\mathop \sum \nolimits_{u,v} {x}_{uv}( {\widehat {{j}_c}} )}}.
\end{eqnarray*}$$

Then, we denoted $\vec{\pi } = {\{ {{\pi }_{mn}( {\widehat {{j}_c}} )} } \},m,n = 1, \cdots ,G,$ where $G$ represents the number of all genomic fragments. $\vec{\pi }$ is assumed to be a mixture of background interactions and biological interactions. We don't deconvolute them explicitly to keep the experimental characteristics of the raw data (Zheng and Kele, 2020).

Specifically, the FIs to be sampled are split into two types: trans-chromosome FIs and cis-chromosome FIs. The frequencies of trans-chromosome FIs are denoted as ${\vec{\pi }}_{{\rm{trans}}}$, and the frequencies of cis-chromosome FIs, denoted as ${\vec{\pi }}_{\rm cis}$, are divided into different genomic distances to preserve the heterogeneity of each cell. The distance-stratified sampling indeed enhances the ability of scHi-CSim to maintain heterogeneity of the raw scHi-C data (Figure 2B; Supplementary Figures S2A and S3). In order to sample separately with different genomic distances, we divide the ${\vec{\pi }}_{\rm cis}$ into $K$ categories as $ {\{ {{{\vec{\pi }}}_{\rm cis}( 1 ), \cdots ,{{\vec{\pi }}}_{\rm cis}( k ), \cdots , {{\vec{\pi }}}_{\rm cis}( K )} } \}$.

For simulated cell $j$, we have ${\rm{fcd}}{( k )}_j$, ${P}_{{\rm{trans}}}( j )$, ${P}_{\rm cis}( j )$, and $f( j )$. The number of FIs belong to trans-chromosome is denoted as ${f}_{{\rm{trans}}}( j )$. We sample ${f}_{{\rm{trans}}}( j )$ FIs by trans-chromosome FI frequencies as ${X}_{{\rm{trans}}}( j )$. For each ${\rm{lo}}{{\rm{g}}}_2$ genomic bin $K$, the number of FIs is $f( {k,j} )$. Then, we sample $f( {k,j} )$ FIs by cis-chromosome FI frequencies as ${X}_{\rm cis}( {k,j} )$ and traverse all $k$s. The final simulated cell is denoted as $X( j )$, the union of ${X}_{\rm cis}$ and ${X}_{{\rm{trans}}}$. We sample all FIs without replacement in order to approximate the practice of scHi-C sequencing technology. After repeating the three steps for each cell, the simulated data are generated by scHi-CSim and the corresponding format is fragment interactions.

### Code availability

Our data and code of scHi-CSim are available at: https://github.com/zhanglabtools/scHi-CSim.

## Supplementary Material

mjad003_Supplemental_File
