# Supplementary material for: scHi-CSim: a flexible simulator that generates high-fidelity single-cell Hi-C data for benchmarking
Source: J Mol Cell Biol. 2023 Jan 27;15(1):mjad003. doi: 10.1093/jmcb/mjad003 (PMC10308180; doi:10.1093/jmcb/mjad003)
Supplement: mjad003_Supplemental_File [file mjad003_supplemental_file.pdf]

Supplementary Materials for

scHi-CSim: a flexible simulator that generates high fidelity single-cell Hi-C data for benchmarking

Usage of scHi-CSim

The source codes of scHi-CSim and detailed instructions can be obtained from https://github.com/zhanglabtools/scHi-CSim. The pipeline of scHi-CSim consists of three parts pre-processing, simulation and post-processing.

Pre-processing

The simulating process of scHi-CSim needs to merge nearby cells to overcome the problems caused by single-cell sparsity. Therefore, the goal of pre-processing is to generate cell-cell distances for finding nearby cells. Here we provide scripts for extracting features using contact probability distribution versus genomic distance (CDD) and pairs' contact coverage (PCC). These features of single-cell Hi-C data is used for estimating the distances between any two cells and constructing a cell-neighbor graph. The calculation of cell-cell distances is performed with two scripts:

```
python extract_features.py -p parameters.txt -b 10
```

```
python calculate_cell_cell_distance.py -p parameters.txt -c 2
```

Then the cell-cell distance file is generated and placed in the "features" directory. Both the scripts need to provide parameters.txt, a file located in the root directory of scHi-CSim, containing essential information of input Hi-C data. The user can also provide user-defined cell-to-cell distances as a file, put it in the "features" folder, and name it "cell_cell_distance.txt". It is recommended to use CIRCLET (https://github.com/zhanglabtools/CIRCLET) to construct the distance relationship between cells for circular cell trajectories [3].

Simulation

Then, scHi-CSim simulates the raw cells by running the following script:

```
python simulating.py -p parameters.txt
```

scHi-CSim is flexible in specifying the number of interactions and replicates of simulated cells by tuning them in the "parameters.txt" file.

Post-processing

Finally, the data generated by the simulation can be combined and converted into bin pairs data by running two scripts:

```
python merge_cell.py -p parameters.txt -m data\merge_data\merge_cell_name_list.txt -i data\sim_data -o data\merge_data
```

```
python convert_chr_pos_to_bin.py -p parameters.txt -i combine_data\chr_pos -o combine_data\bin_pairs -r resolution
```

The resolution is assigned by r, and the generated sparse bin pairs data can be used for downstream analysis, such as detection of loops and TADs and clustering.

Parameters setting

Supplementary Table S2 displays the parameters used in scHi-CSim. For more details, please refer to the documentation (https://github.com/zhanglabtools/scHi-CSim)

Complexity and running time

The complexity of **Step 1** is $O(n_1)$, where n_1 represents the total number of fragment interactions in raw data. The complexity of **Step 2** and **Step 3** are $O(n_2)$, where n_2 represents the total number of fragment interactions in simulated data. Besides, $Step2$ and $Step3$ are performed independently for each cell. Therefore, they are highly scalable for parallel computation. In summary, the complexity of scHi-CSim is $O(n)$, where $n = \max(n_1, n_2)$ and the usage of a multi-kernel CPU will significantly accelerate the simulation process.

Supplementary Fig. 15 shows the usage of time in different sequencing depth with core 1, 4, 8, 12, 24. When the numbers of cores are 1, 4, 8, 12, 24, the peak memory usages of these experiments are 736Mb, 3,674Mb, 10,678Mb, 13,669Mb, and 21,849Mb, respectively.

Methods

Jaccard index

Jaccard index is used to gauge the similarity of two sets. We define

$$J(A,B) = \frac{\mathrm{lvert}\, A \cap B\, \mathrm{rvert}}{\mathrm{lvert}\, A \cup B\, \mathrm{rvert}},$$

where A and B are two sets, such as TAD sets. The higher the Jaccard index, the more similarity between A and B.

Pearson correlation coefficient

Pearson correlation coefficient is defined as

$$\rho_{X,Y} = \frac{\mathrm{cov}(X,Y)}{\sigma_X \sigma_Y},$$

where X and Y are random variables, cov is the covariance, σ_X and σ_Y are the standard deviation of X and Y respectively.

Compartments detection

Compartments are detected by CscoreTool [4]. CscoreTool assume that P_i is the chance of each genomic window i belonging to be in the A-compartment and define C-score as$C_i = 2P_i - 1$, which ranges between -1 and 1. Then perform the maximum-likelihood estimation for the following log-likelihood function:

$$lnL(B,C,H) = \sum_{i<j} \{ n_{ij} \ln [B_i B_j H(d_{ij})(1 + C_i C_j)] - B_i B_j H(d_{ij})(1 + C_i C_j) \},$$

where n_{ij} represents the observed number of contacts, d_{ij} represents the genomic distance, $H(d_{ij})$ represents the scaling factor accounting for the decay profile of longer interactions, and

PCR biases or genome mappability of Hi-C experiments is described as B_i and B_j. Finally, C-score vectors will represent the division of genomic.

TADs detection

TADs are detected by Insulation Score [1]. Hi-C data is first converted to a bin-separated matrix according to a fixed resolution, such as 10kb, 50kb, and so on. A fixed width square is slid along the Hi-C matrix's main diagonal that generates insulation square, the mean contacts of the bins belonging to the square. Next, insulation delta calculation is denoted as $\text{delta} = \text{mean}(\text{left}) - \text{mean}(\text{right})$ for each bin. The bin where the insulation score is a local minimum and the insulation delta is equal to 0 is defined as the TAD boundaries. Besides, for the boundaries' quantitative analysis, the difference between the local maximum and minimum of the insulation delta per boundary is defined as insulation strength. The higher the insulation strength, the greater the difference between the two sides of the boundary.

Percentage

In order to compare the loops between the raw and simulated data, we define percentage as

$$\text{Percentage} = \frac{\text{lvertLoop}(\text{Top}N)_{\text{raw}} \cap \text{Loop}(\text{Top}N)_{\text{simulated}}\text{rvert}}{\text{lvertLoop}(\text{Top}N)_{\text{raw}}\text{rvert}},$$

where $N = 100, 200, \cdots, 2000$.

Hypergeometric test

The probability mass function for the hypergeometric test is given by

$$\text{Pr}(k) = \frac{\text{tbinom}Kk\,\text{tbinom}M-Kn-k}{\text{tbinom}Mn},$$

where M represents the number of background genes (all genes of the mouse), n represents the number of genes overlapping with top N differentials loops, K and k represent the total number and the observed number of cell-cycle annotated genes or DEGs.

Circular normal distribution

The circular normal probability density function for the angle x is defined as:

$$f(x \mid \mu, \kappa) = \frac{e^{\kappa \cos(x-\mu)}}{2\pi I_0(\kappa)},$$

where $I_0\kappa$ is the modified Bessel function of order 0, μ and κ are the location and concentration, respectively.

CROC and ACROC

To evaluate the cell-cycle data, the embedding of each stage is assumed to obey circular normal distribution [2]. Then we calculated CROC score and ACROC after fitting each stage to a circular normal distribution. More formally, the embedding results assigned angles along the cell cycle for n cells as $(\theta_1, \ldots, \theta_n)$, and the labels of n cells is denoted as $(l_1, \ldots, l_n)$. For each cell, naturally $0 \le \theta_i \le \pi$ and $1 \le l_i \le m$ in which m represent the number of labels types. In order to calculate the CROC score, we first set one label as positive class and the others as negative class, and denote the mean angle as θ^* by fitting the cells of one stage to a circular normal distribution. Then we design the absolute difference between one cells' angle and the mean angle as:

$\hat{\theta}_i = \min(|\theta_i - \theta^*|, |\theta_i - 2\pi - \theta^*|)$. Then AUC of one stage (CROC score) is calculated according to the $\hat{\theta}_i$ and the class of each cell. Four CROC scores under the four stages were averaged to get ACROC.

ARI

Adjusted Rand index (ARI) is used to measure the similarity between predicted classes and true classes. We define

$$\mathrm{ARI} = \frac{\sum_{ij}\binom{n_{ij}}{2} - \left[\sum_i \binom{a_i}{2}\sum_j \binom{b_j}{2}\right] / \binom{n}{2}}{\frac{1}{2}\left[\sum_i \binom{a_i}{2} + \sum_j \binom{b_j}{2}\right] - \left[\sum_i \binom{a_i}{2}\sum_j \binom{b_j}{2}\right] / \binom{n}{2}},$$

where ARI is based on confusion matrix N, n_{ij} represents the cells number that belongs to i th cell type labeled by biological marks and j th cluster predicted by the algorithm, a_i and b_i are corresponding to the total number of the i th row and the j th column of N, respectively, and n is the total number of cells.

Reference

[1] Emily Crane, Qian Bian, Rachel Patton McCord, Bryan R Lajoie, Bayly S Wheeler, Edward J Ralston, Satoru Uzawa, Job Dekker, and Barbara J Meyer. Condensin-driven remodelling of x chromosome topology during dosage compensation. *Nature*, 523(7559):240–244, 2015.

[2] Jie Liu, Dejun Lin, Galip Gürkan Yardmc, and William Stafford Noble. Unsupervised embedding of single-cell hi-c data. *Bioinformatics*, 34(13):i96–i104, 2018.

[3] Yusen Ye, Lin Gao, and Shihua Zhang. Circular trajectory reconstruction uncovers cell-cycle progression and regulatory dynamics from single-cell hi-c maps. *Advanced Science*, 6(23):1900986, 2019.

[4] Xiaobin Zheng and Yixian Zheng. Cscoretool: fast hi-c compartment analysis at high resolution. *Bioinformatics*, 34(9):1568–1570, 2018.

Supplementary Table S1

ARI of clustering results on simulated data.

Using PCA for constructing cell neighbor graph (ARI)			
Datasets	Clustering methods	Not using cell type information	Using cell type information
Ramani et al. dataset	PCA	0.588	0.602
	scHiCluster	0.762	0.534
	HiCRep/MDS	0.238	0.246
	LDA	0.730	0.389
4DN sci-Hi-C dataset	PCA	0.171	0.205
	scHiCluster	0.287	0.638
	HiCRep/MDS	0.182	0.176
	LDA	0.290	0.724

Using scHiCluster for constructing cell neighbor graph (ARI)			
Datasets	Clustering methods	Not using cell type information	Using cell type information
Ramani et al. dataset	PCA	0.579	0.602
	scHiCluster	0.613	0.632
	HiCRep/MDS	0.268	0.231
	LDA	0.741	0.718
4DN sci-Hi-C dataset	PCA	0.127	0.280
	scHiCluster	0.516	0.519
	HiCRep/MDS	0.180	0.173
	LDA	0.438	0.718

Using HiCRep/MDS for constructing cell neighbor graph (ARI)			
Datasets	Clustering methods	Not using cell type information	Using cell type information
Ramani et al. dataset	PCA	-0.003	0.558
	scHiCluster	0.444	0.626
	HiCRep/MDS	0.241	0.238
	LDA	0.310	0.777
4DN sci-Hi-C dataset	PCA	0.107	0.299
	scHiCluster	0.375	0.625
	HiCRep/MDS	0.176	0.176
	LDA	0.331	0.590

Using LDA for constructing cell neighbor graph (ARI)			
Datasets	Clustering methods	Not using cell type information	Using cell type information
Ramani et al. dataset	PCA	0.592	0.563
	scHiCluster	0.403	0.423
	HiCRep/MDS	0.238	0.233
	LDA	0.390	0.252
4DN sci-Hi-C dataset	PCA	0.261	0.239
	scHiCluster	0.608	0.625
	HiCRep/MDS	0.181	0.175
	LDA	0.699	0.695

Supplementary Table S2
Primary parameters in scHi-CSim.

Parameter Name	Default Value	Description
combineNumber	20	The number of merged cells
step	0.04	The step size for dividing chromosomes
bin_interval_number	200	Number of intervals when stratified sampling
parallel	True	Turn on parallel computing
kernel_number	24	Set the number of cores
filter_distance	1000000	The threshold of chromosomal distances for filtering noisy signals
filter_value_percentile	20	The percentile of values for filtering noisy signals.

Supplementary Table S3

The number of cells in the three scHi-C datasets.

Dataset	Cell type/Cell state	Count
Ramani et al.	K562	92
	HeLa	252
	HAP1	230
	GM12878	39
	**Total**	613
4DN sci-Hi-C	H1Esc	1158
	HAP1	1460
	HFF	382
	IMR90	34
	GM12878	2073
	**Total**	5107
Nagano et al.	G1	280
	early-S	303
	late-S/G2	326
	mid-S	262
	**Total**	1171

Supplementary Figure S1

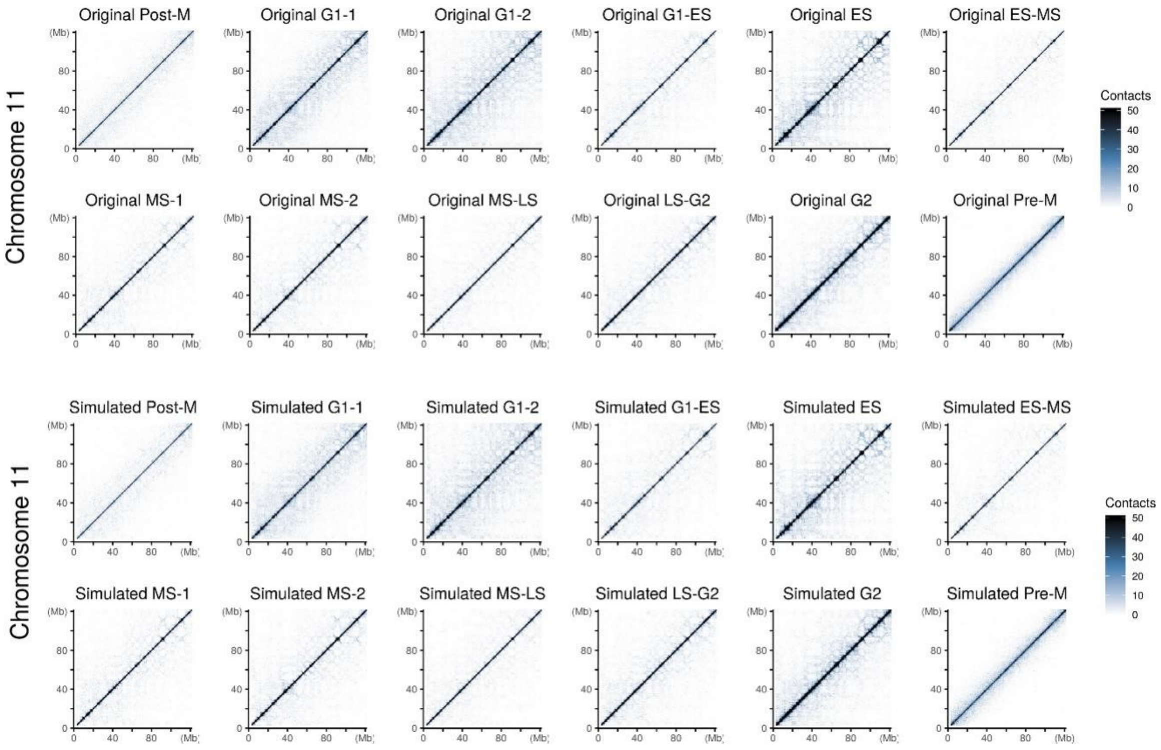

Supplementary Figure S1. The pooled Hi-C contact matrices of 12 cell-cycle stages inferred by CIRCLET of the raw data and the scHi-CSim simulated ones.

Supplementary Figure S2

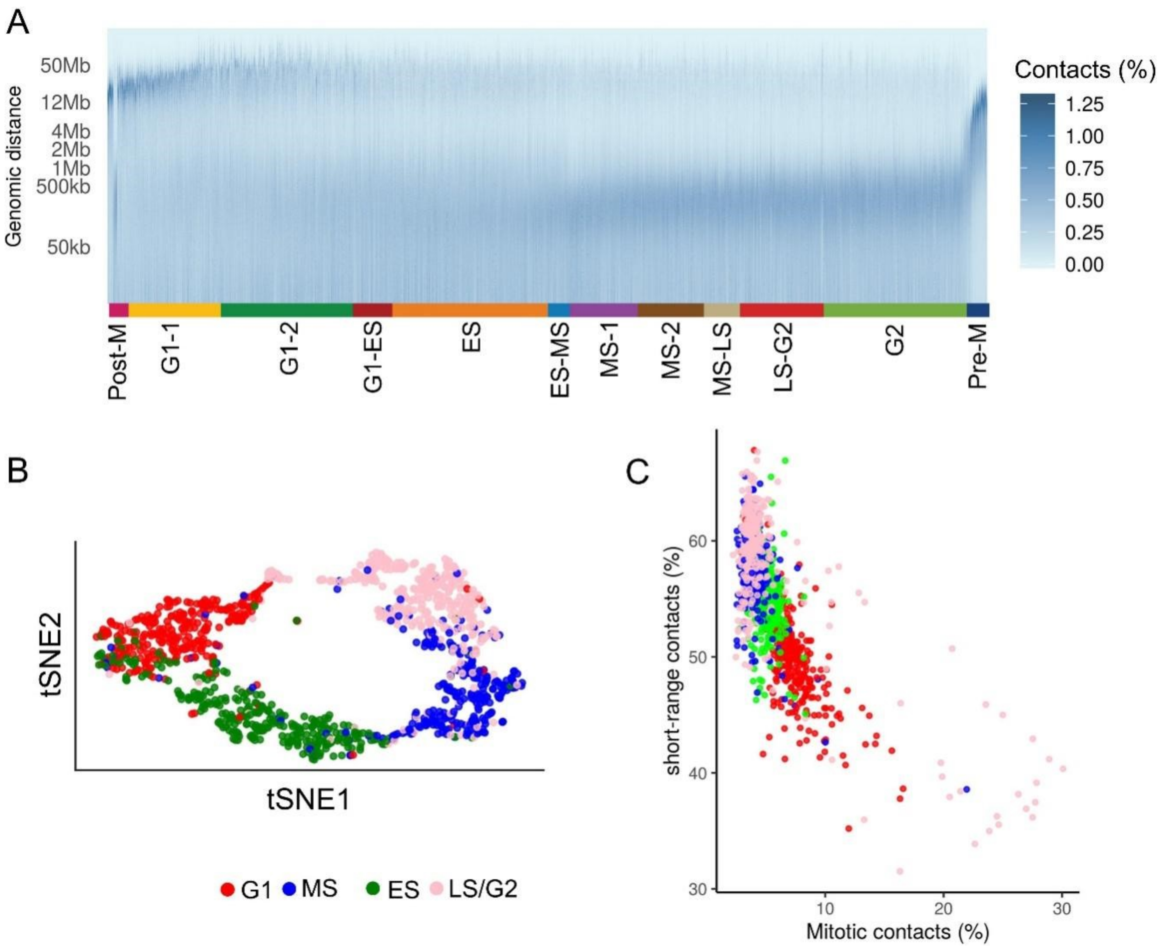

Supplementary Figure S2. Properties of raw 1171 single-cell hESC Hi-C data. (A) raw single-cell contact decay profiles ordered by CIRCLET inferred cell-cycle phasing shown on the bottom. Each column represents a single cell. (B) tSNE maps of raw single cells calculated by CIRCLET based on the combination of feature sets CDD and PCC from four FACS-sorted cell phased (G1, ES, MS, and LS/G2). (C) Percentage of short-range (<2 Mb) versus mitotic band (2-12 Mb) contacts per raw cell. Cells are coloured by four FACS-sorted cell phases (G1, ES, MS, and LS/G2).

Supplementary Figure S3

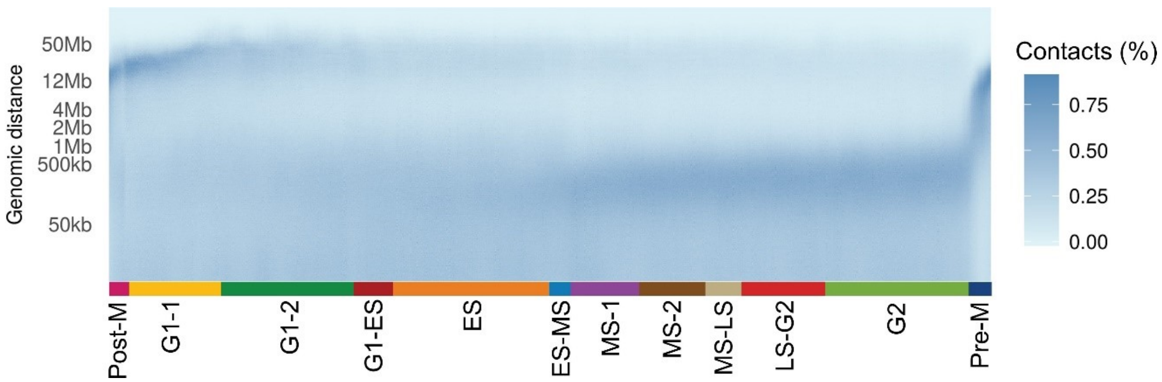

Supplementary Figure S3. The decay profiles of scHi-CSim simulated cells without using distance-stratified sampling.

Supplementary Figure S4

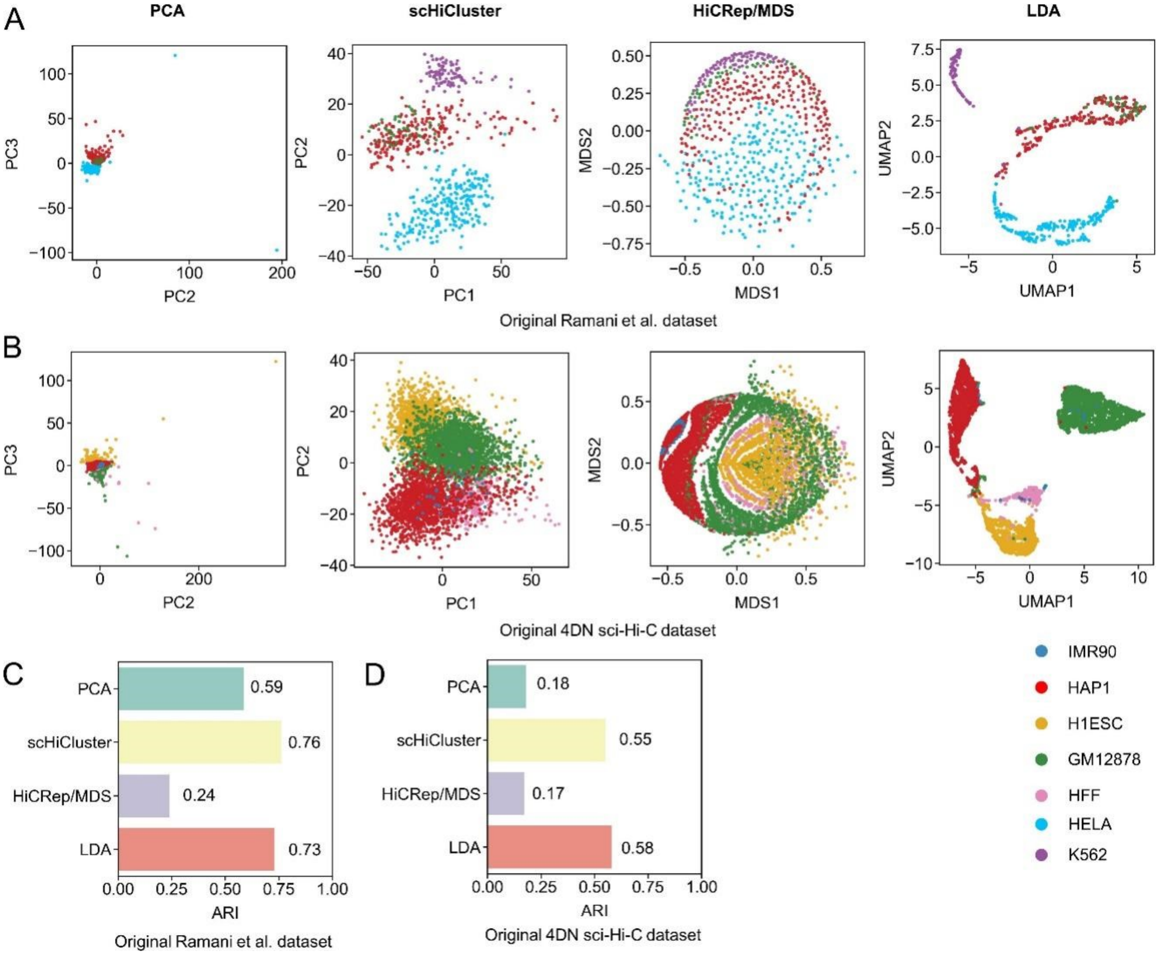

Supplementary Figure S4. The clustering results of the original Ramani et al. dataset and 4DN sci-Hi-C dataset. (A, B) The embedding results of four clustering methods on Ramani et al. dataset (A) and 4DN sci-Hi-C dataset (B). (C, D) ARI of clustering results on Ramani et al. dataset (C) and 4DN sci-Hi-C dataset (D).

Supplementary Figure S5

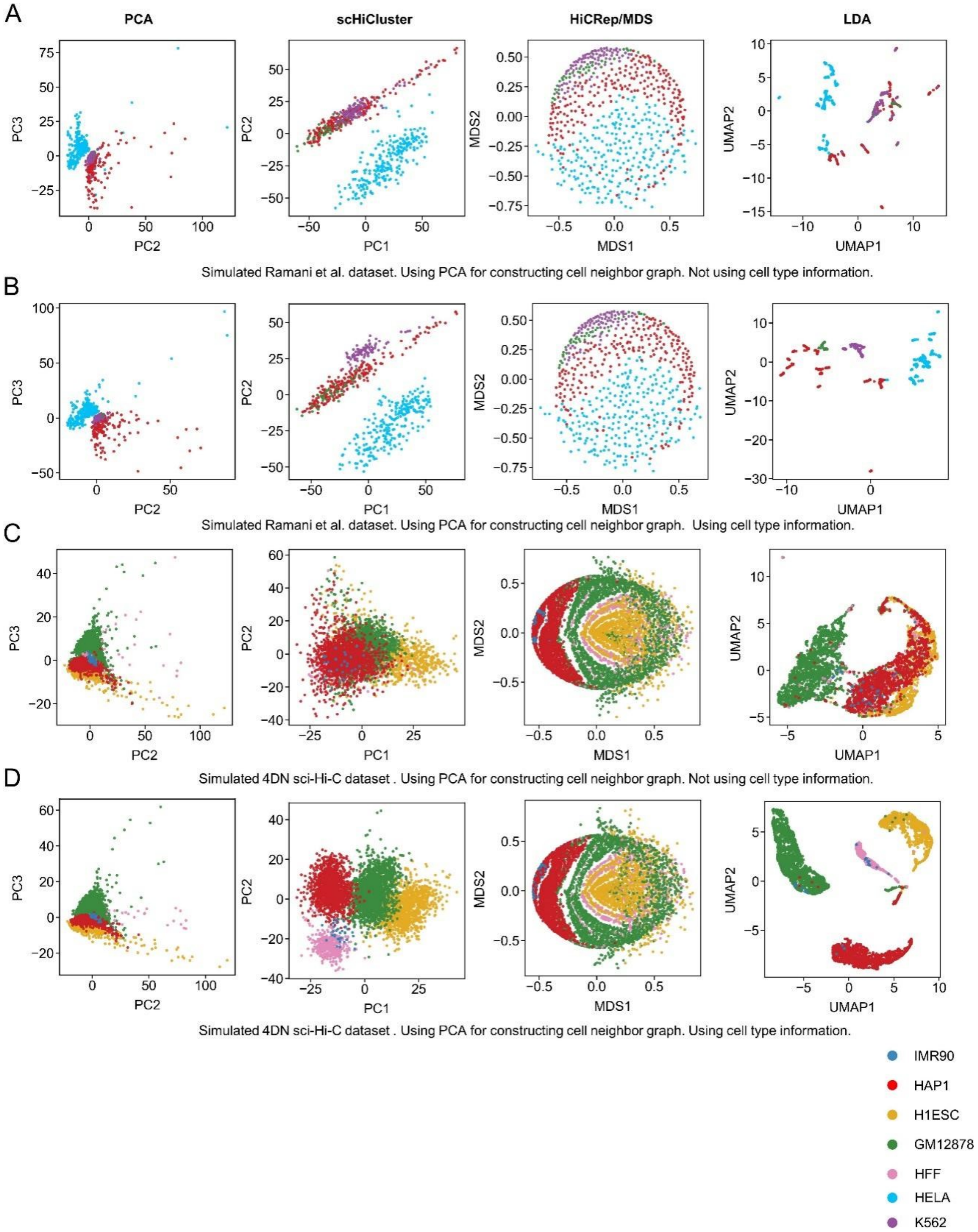

Supplementary Figure S5. The simulated data using PCA for constructing cell neighbor graphs.

Supplementary Figure S6

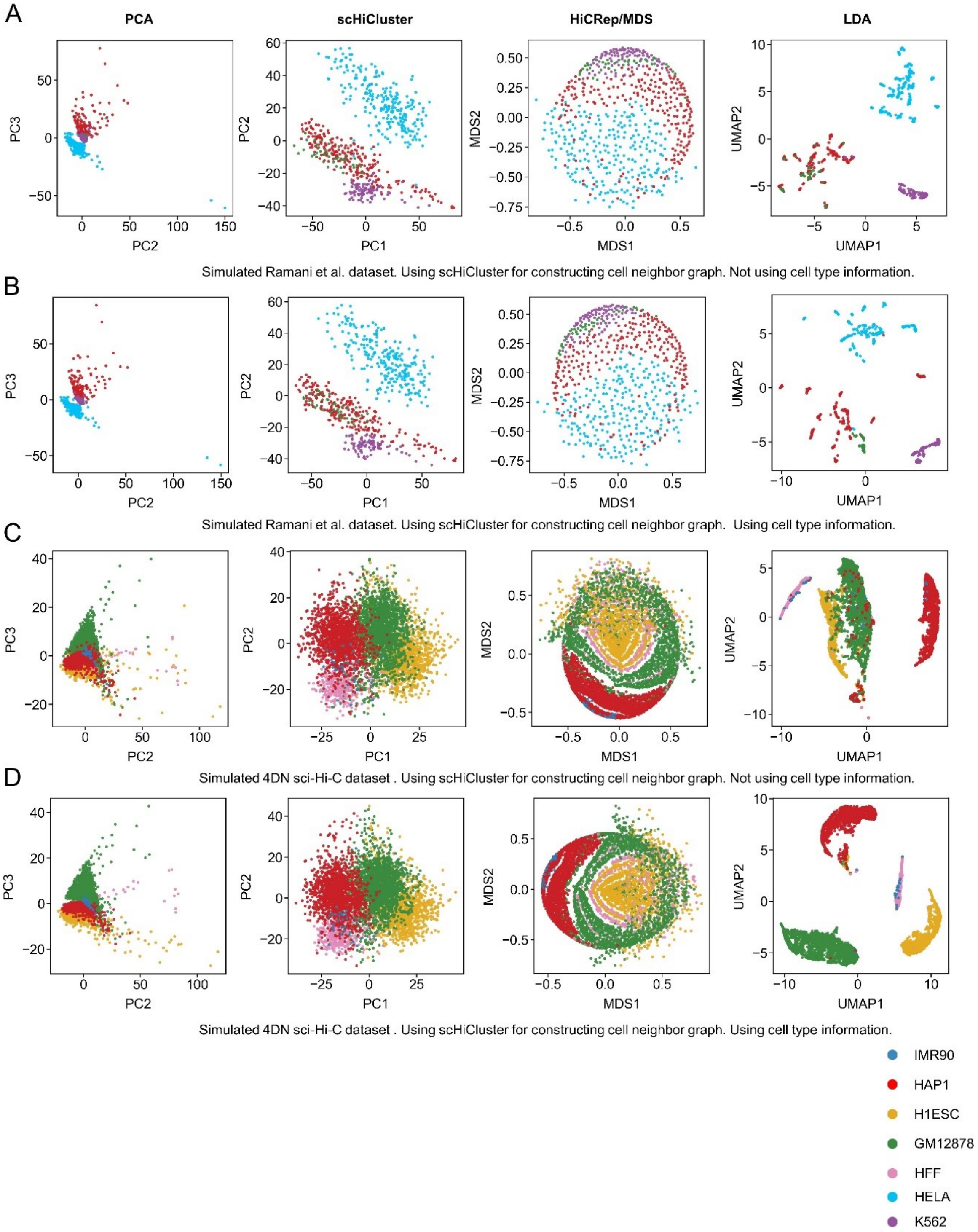

Supplementary Figure S6. The simulated data using scHiCluster for constructing cell neighbor graphs.

Supplementary Figure S7

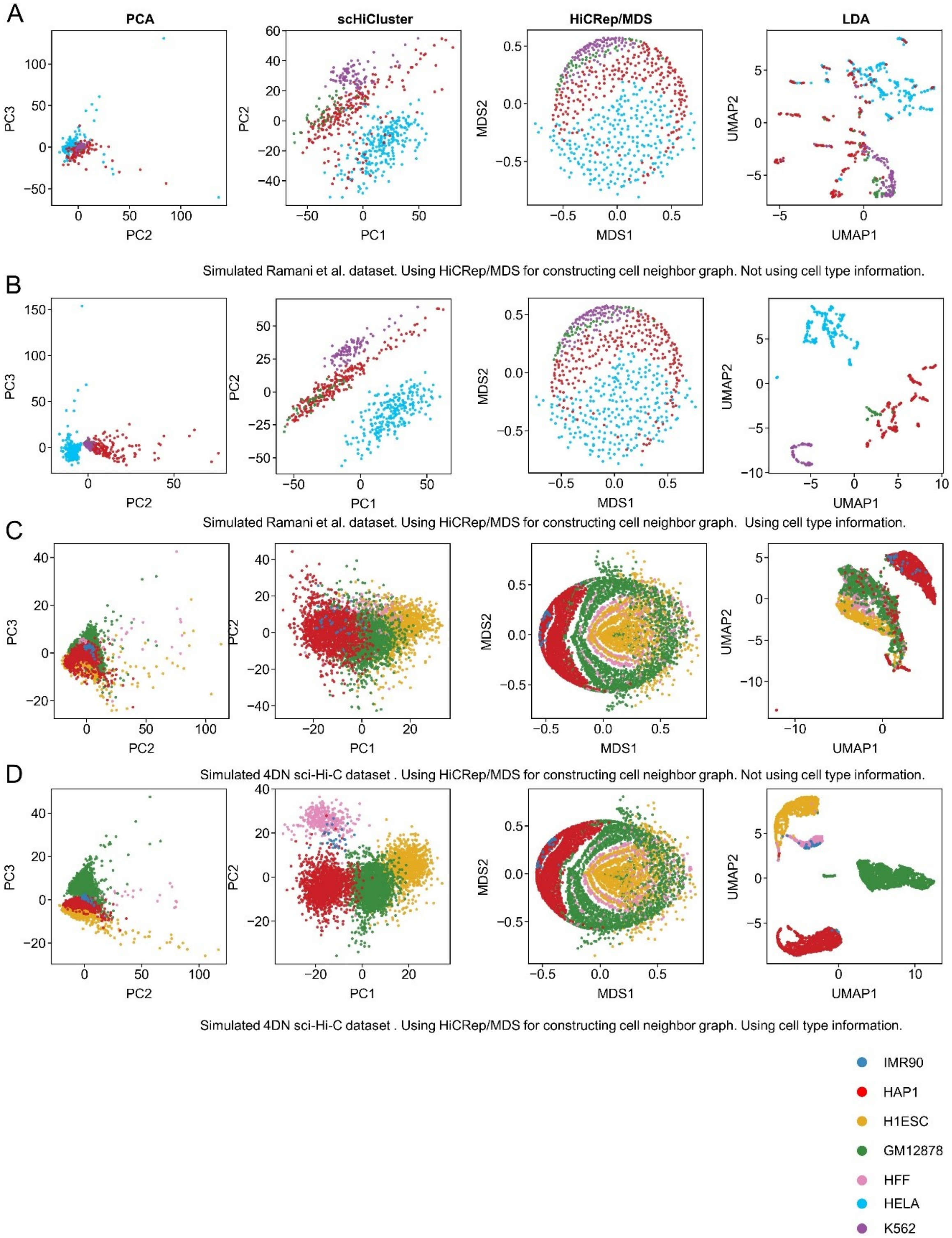

Supplementary Figure S7. The simulated data using HiCRep/MDS for constructing cell neighbor graphs.

Supplementary Figure S8

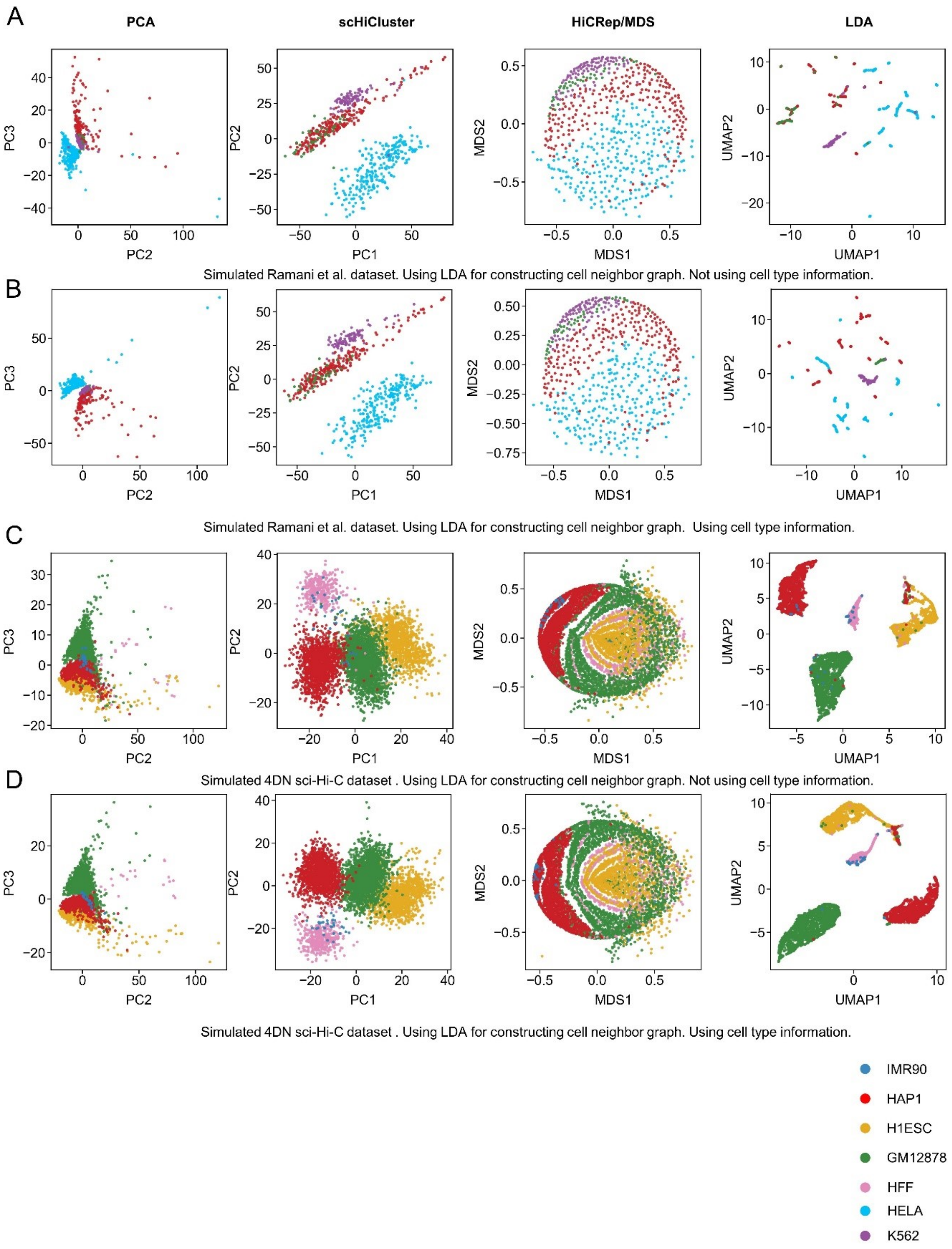

Supplementary Figure S8. The simulated data using LDA for constructing cell neighbor graphs.

Supplementary Figure S9

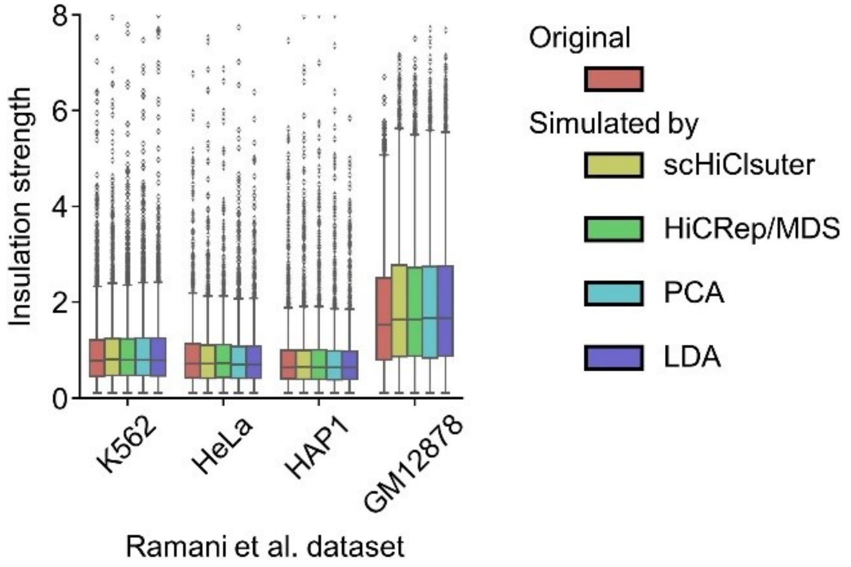

Supplementary Figure S9. Insulation strength of TADs' boundaries. TAD boundaries are detected using the Insulation Score (25 kb).

Supplementary Figure S10

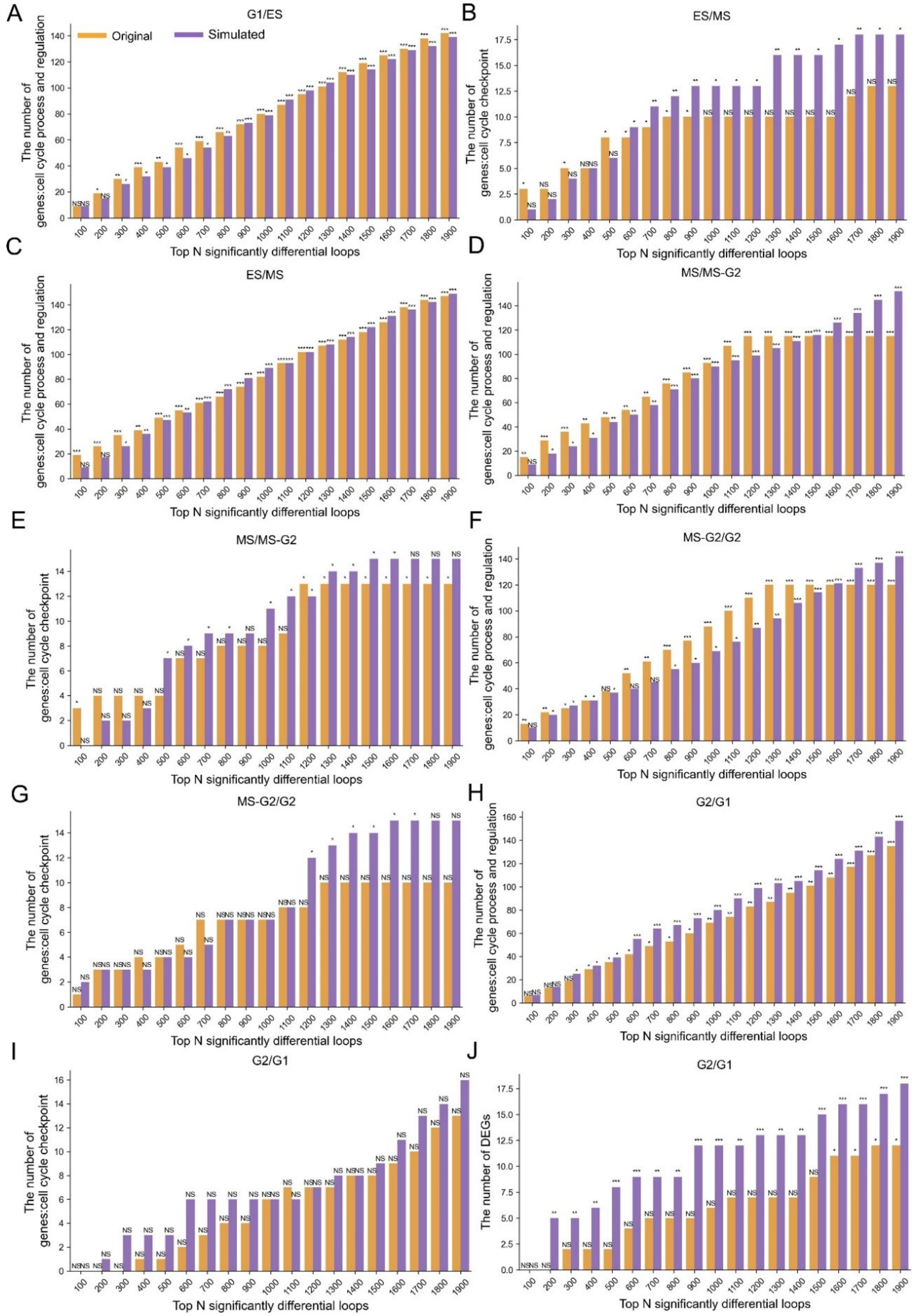

Supplementary Figure S10. Number of genes in top N significant differential loops of raw data and scHi-CSim simulated data and hypergeometric testing for significant analysis of genes come from cell cycle checkpoint, cell cycle process and regulation and differentially expressed genes. (A) Number of genes associated with cell cycle process and regulation in differential loops between G1/ES. (B, C) Number of genes associated with cell cycle checkpoint (B), cell cycle process and regulation (C) in differential loops between ES/MS. (D, E) Number of genes associated with cell cycle checkpoint (E), cell cycle process and regulation (D) in differential loops between MS/MS-G2. (F, G) Number of genes associated with cell cycle checkpoint (G), cell cycle process and regulation (F) in differential loops between MS-G2/G2. (H, I, J) Number of genes associated with cell cycle checkpoint (I), cell cycle process and regulation (H), differentially expressed genes (DEGs) in differential loops (J) between G2/G1. (NS, Not significant, $P > 5 \times 10^{-2}$, *$P < 5 \times 10^{-2}$, **$P < 5 \times 10^{-3}$, ***$P < 5 \times 10^{-4}$).

Supplementary Figure S11

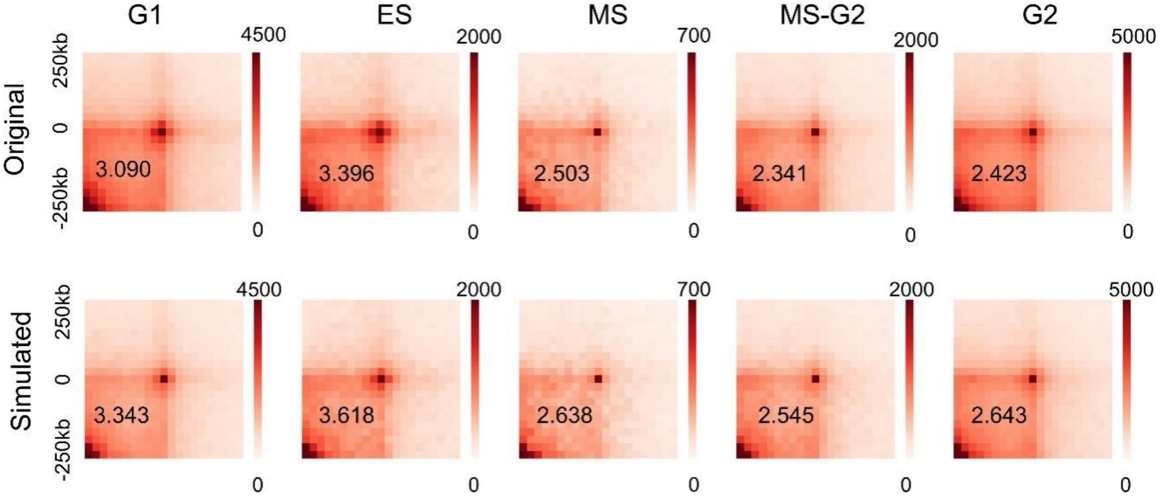

Supplementary Figure S11. Aggregate Peak Analysis (APA) of common loops in raw and simulated data. P2LL (Peak to Lower Left), the ratio of central pixel to the mean of pixels in the lower left corner, is exhibited in the lower left per heatmap.

Supplementary Figure S12

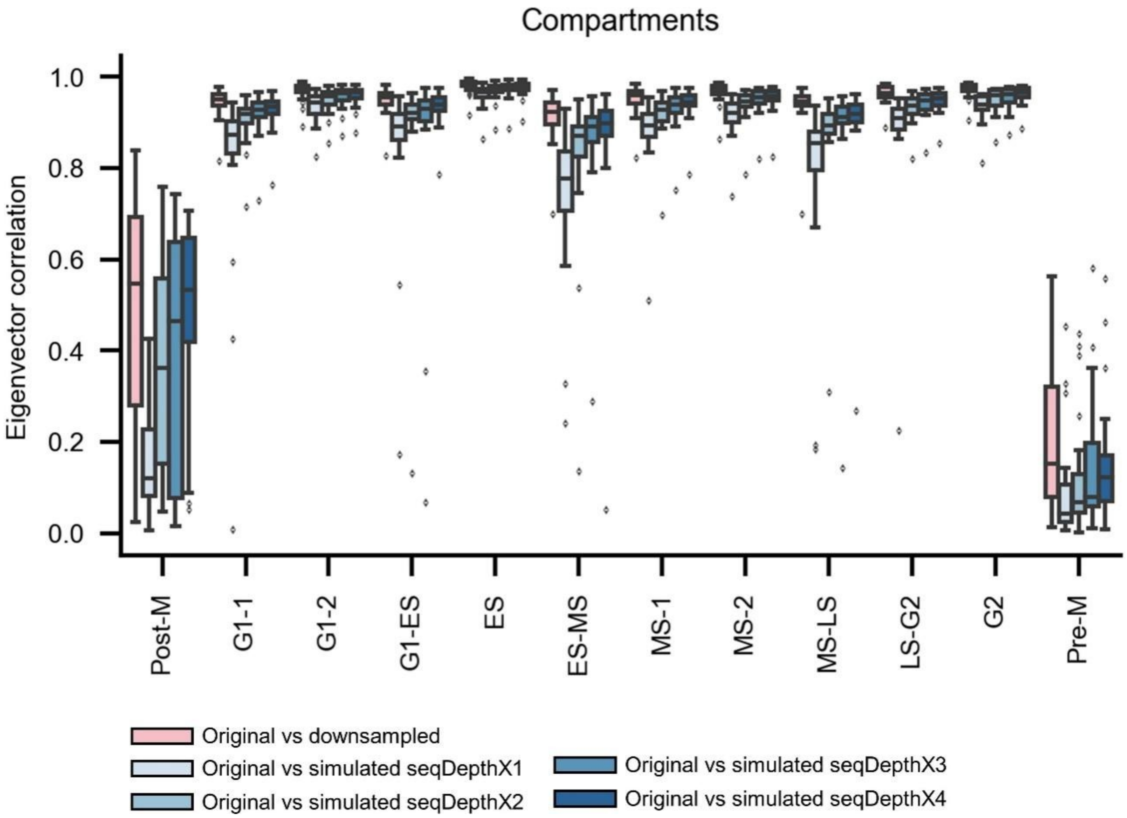

Supplementary Figure S12. Accuracy of detecting compartments (100 kb) with distinct sequencing depths. Eigenvector correlations of the compartments detected using the raw pooled Hi-C maps and the other five datasets: downsampled raw Hi-C maps, simulated Hi-C maps with 1X, 2X, 3X and 4X sequencing depths.

Supplementary Figure S13

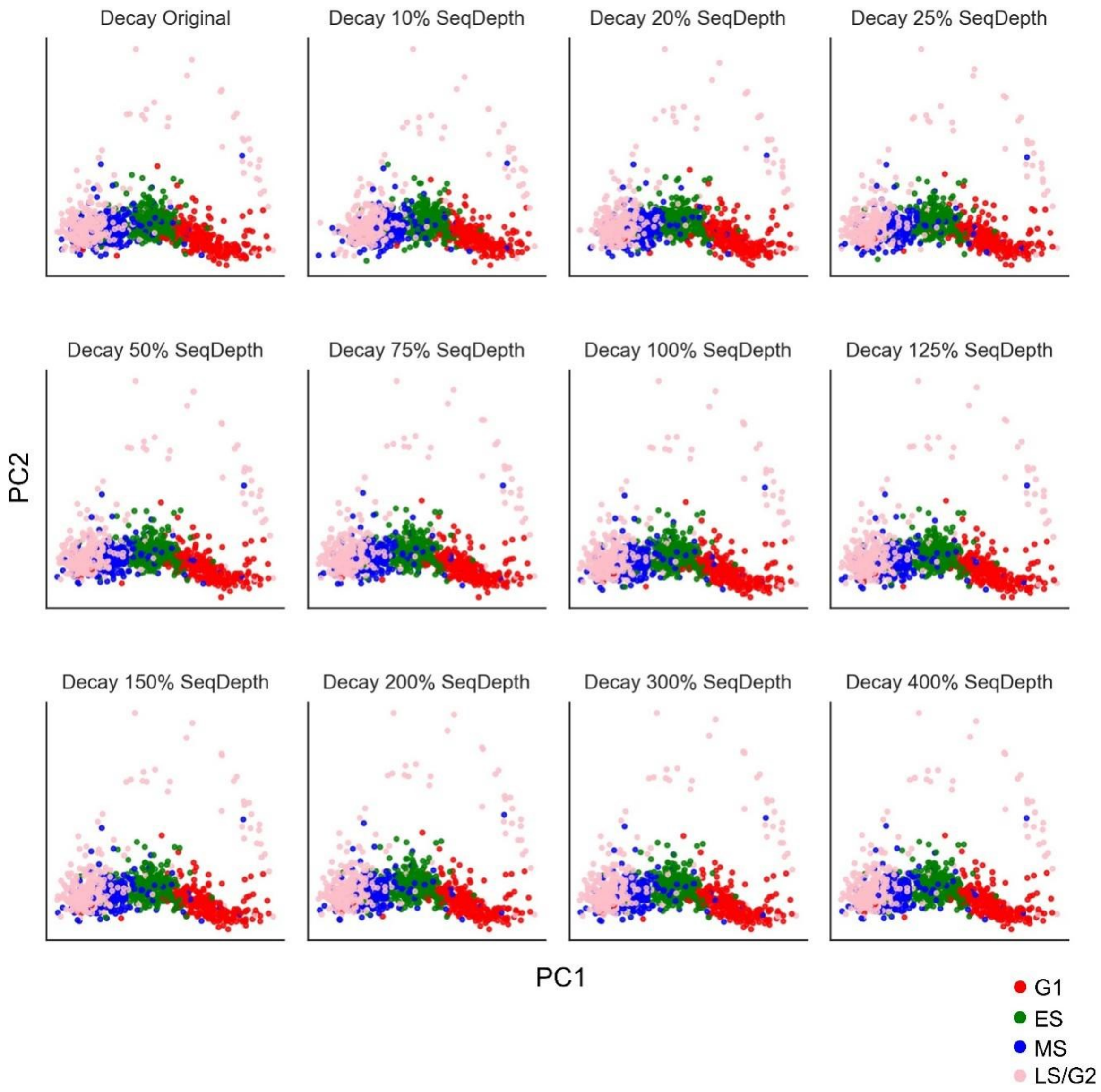

Supplementary Figure S13. Embedding results of Decay (1 Mb).

Supplementary Figure S14

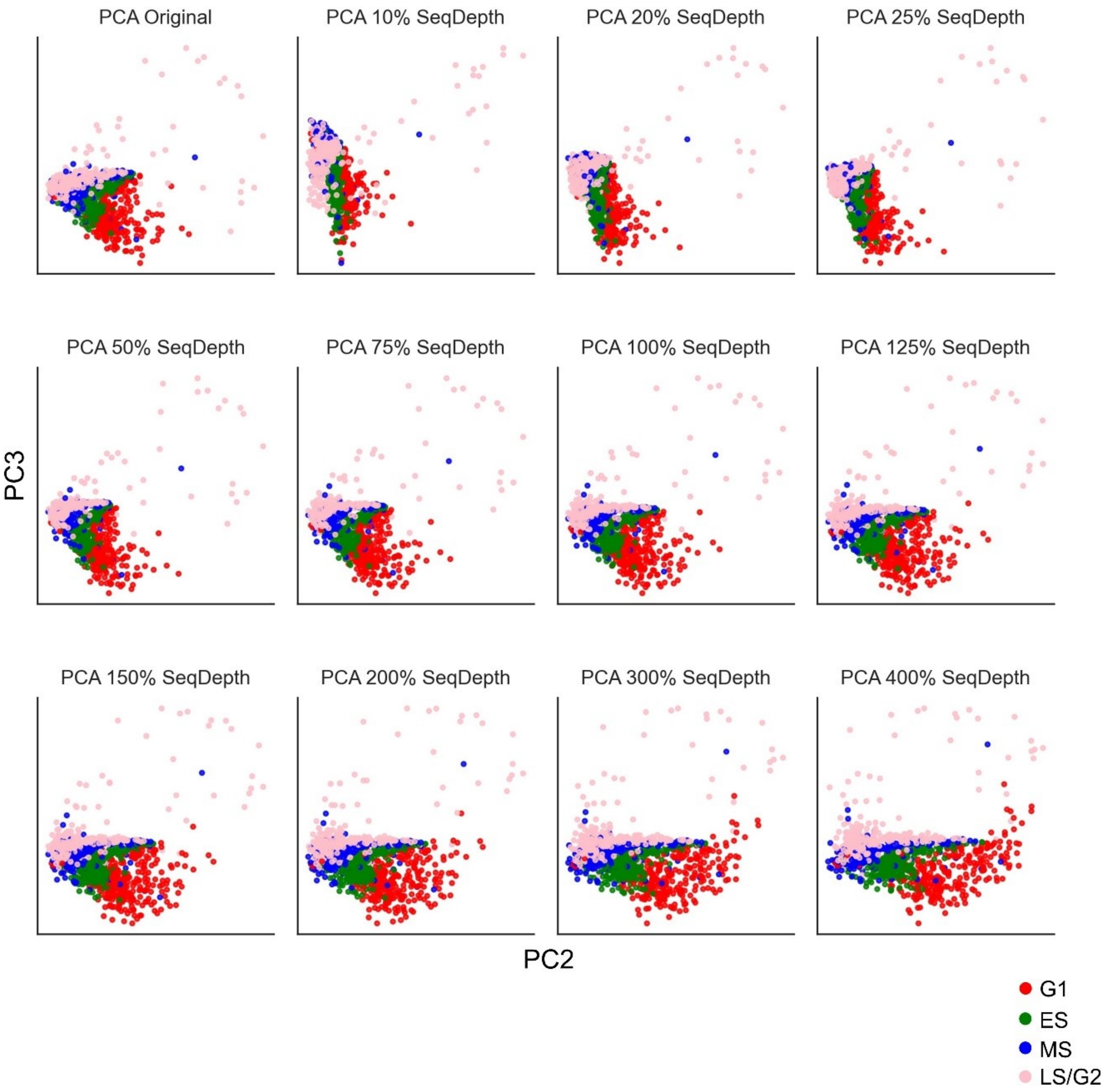

Supplementary Figure S14. Embedding results of PCA (1 Mb).

Supplementary Figure S15

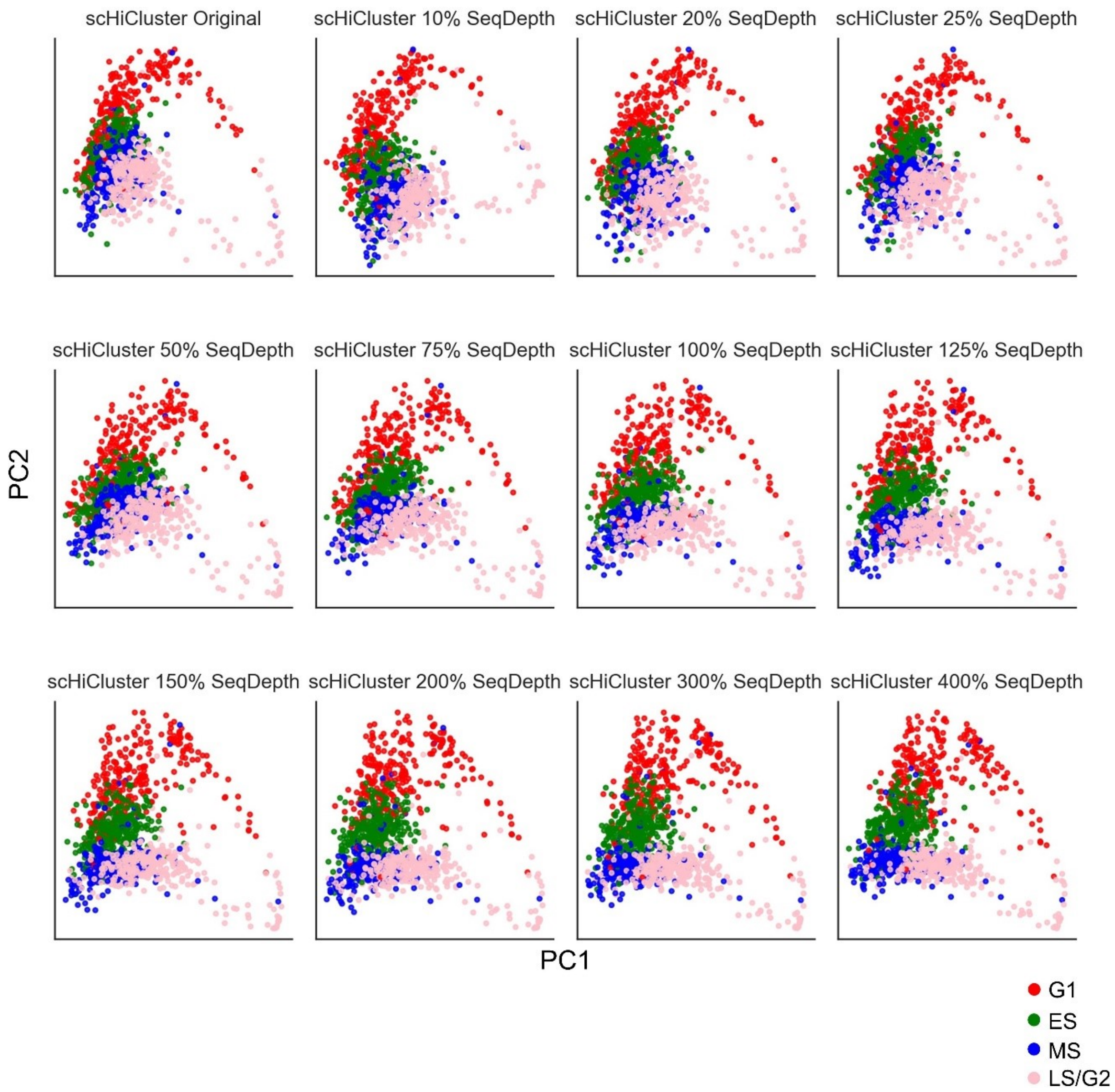

Supplementary Figure S15. Embedding results of scHiCluster (1 Mb).

Supplementary Figure S16

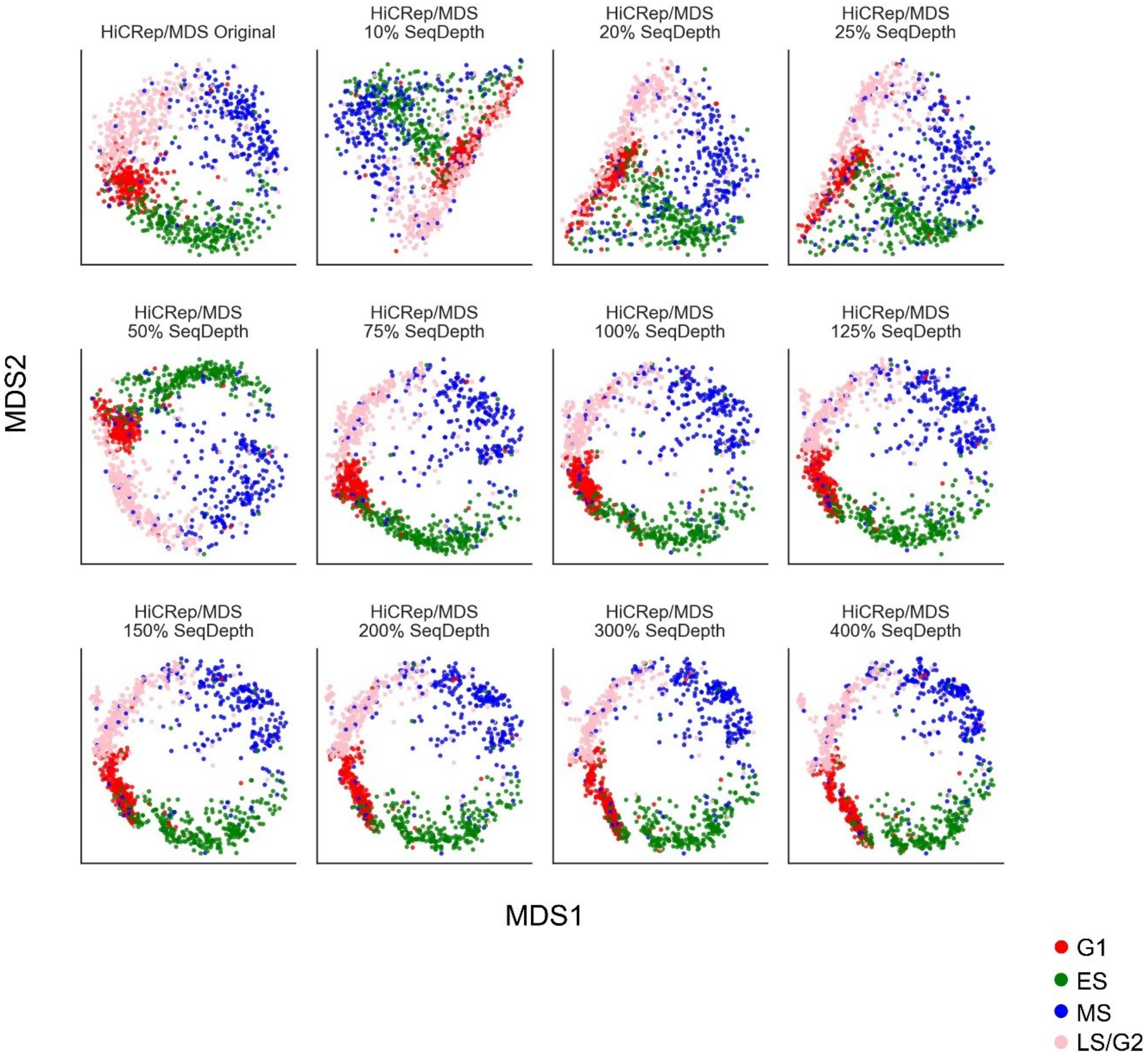

Supplementary Figure S16. Embedding results of HiCRep/MDS.

Supplementary Figure S17

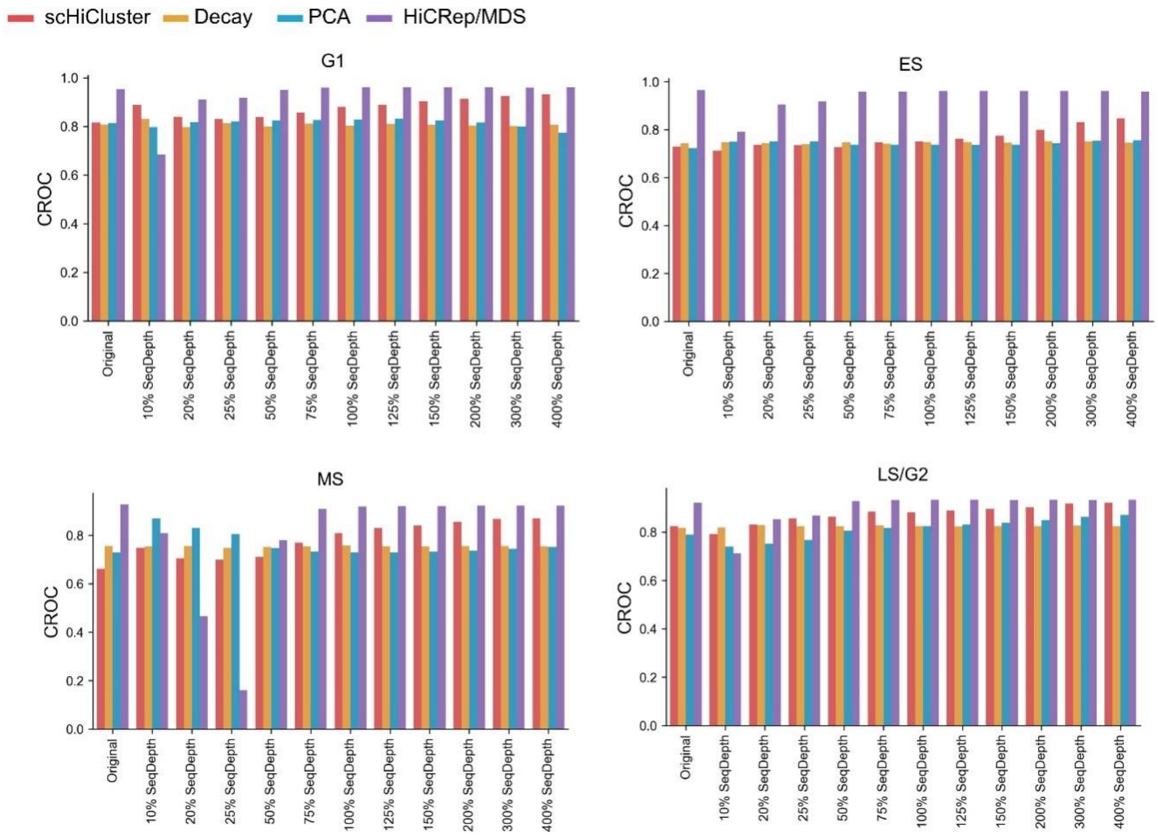

Supplementary Figure S17. CROC of different methods on G1, ES, MS, LS/G2.

Supplementary Figure S18

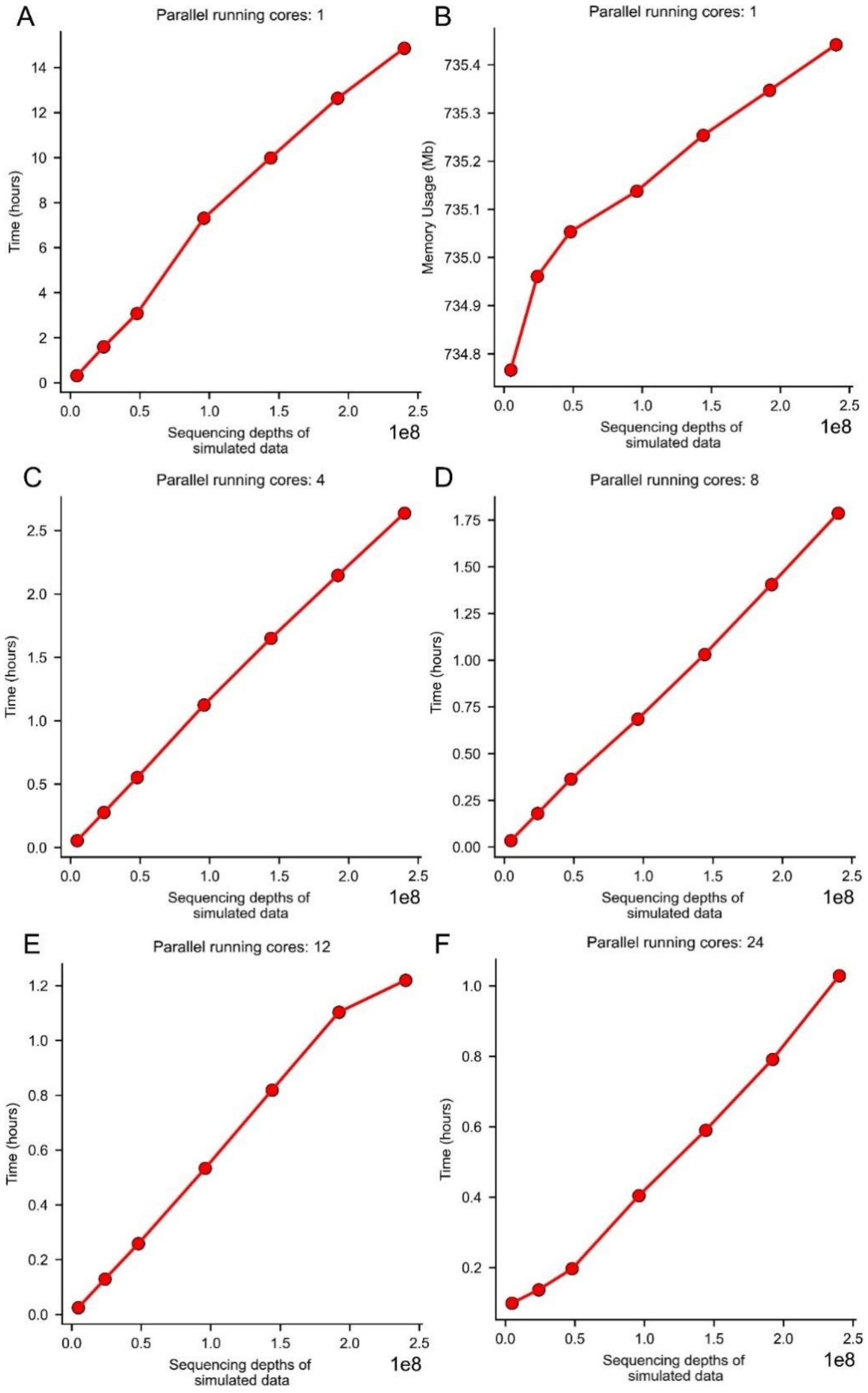

Supplementary Figure S18. The consumption of time and memory when simulating with different CPU kernels. (A, B) The consumption of time (A) and memory (B) when simulating with one CPU kernel. (C-F) The consumption of time when simulating with 4 (C), 8 (D), 12 (E) and 24 (F) CPU kernels respectively.

Supplementary Figure S19

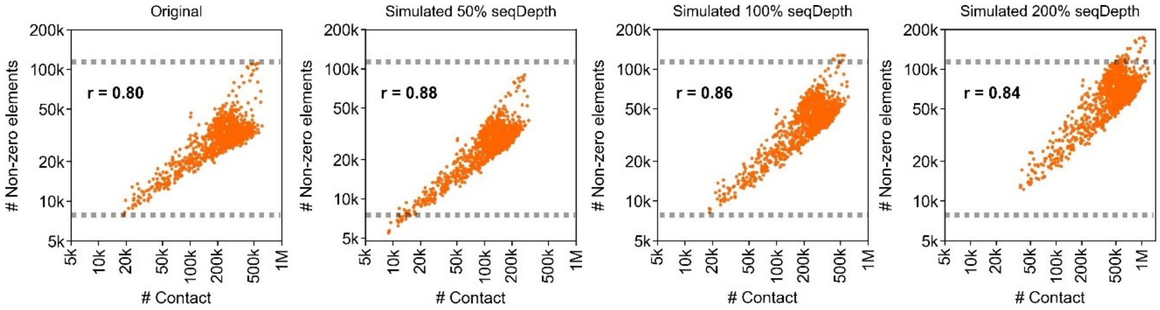

Supplementary Figure S19. The number of contacts and the number of non-zero elements per cell in the original and scHi-CSim simulated data with Nagano et al. dataset (500 kb).
